# Ecological features facilitating spread of alien plants along Mediterranean mountain roads

**DOI:** 10.1007/s10530-024-03418-y

**Published:** 2024-08-08

**Authors:** Lucia Antonietta Santoianni, Michele Innangi, Marco Varricchione, Marta Carboni, Greta La Bella, Sylvia Haider, Angela Stanisci

**Affiliations:** 1 EnviXLab, Department of Biosciences and Territory, University of Molise, Termoli and Pesche, Italy; 2 Department of Science, Roma Tre University, Rome, Italy; 3 Institute of Ecology, Leuphana University of Lüneburg, Lüneburg, Germany; 4 National Biodiversity Future Center (NBFC), 90133 Palermo, Italy

**Keywords:** Alien plants, Mediterranean mountains, Italy, MIREN, Ecological and disturbance indicators

## Abstract

Invasive alien species represent a major threat to global biodiversity and the sustenance of ecosystems. Globally, mountain ecosystems have shown a degree of resistance to invasive species due to their distinctive ecological features. However, in recent times, the construction of linear infrastructure, such as roads, might weaken this resistance, especially in the Mediterranean basin region. Roads, by acting as efficient corridors, facilitate the dispersal of alien species along elevation gradients in mountains. Here, we investigated how the ecological features and road-associated disturbance in native plant communities affected both the occurrence and cover of alien plant species in Central Apennines (Italy). We implemented the MIREN road survey in three mountain transects conducting vegetation sampling in plots located both adjacent to and distant from the roads at intervals of ~ 100 m in elevation. We then used community-weighted means of Ecological Indicator Values for Europe together with Disturbance Indicator Values applied to plant species of native communities as predictors of alien species occurrence and cover in a machine-learning classification and regression framework. Our analyses showed that alien species’ occurrence was greater in proximity to the road where high soil disturbance occurred and in warm- and light-adapted native communities. On the other hand, alien species cover was more strongly related to moderate grazing pressure and the occurrence of nitrophilic plant communities. These findings provide a baseline for the current status of alien plant species in this Mediterranean mountain region, offering an ecological perspective to address the challenges associated with their management under global change.

**Supplementary Information:**

The online version contains supplementary material available at 10.1007/s10530-024-03418-y.

## Introduction

Invasive alien species (IAS) are one of the major threats to global biodiversity (IUCN 2021) and they may have negative socio-economic and human health impacts (Castro-Díez et al. 2019; Pyšek et al. 2020; Henry et al. 2023). In recent times, human activities are facilitating the transport of IAS at unprecedented rates much beyond their native distribution ranges (Van Kleunen et al. 2015; Seebens et al. 2017), and are also promoting range expansion within the introduced ranges, even in protected or highly biodiverse areas (Blackburn et al. 2011; Gioria et al. 2023). To forecast and prevent new invasions and manage existing ones, it is necessary to gain a thorough understanding of the ecological factors that contribute to the successful establishment and spread of IAS, particularly in systems where control measures are still feasible (González-Moreno et al. 2014; Wagner et al. 2017; Langmaier and Lapin 2020; Fuentes-Lillo et al. 2021).

Mountain ecosystems are particularly vulnerable to the combined effects of climate warming and human-mediated disturbances (Schmeller et al. 2022). These drivers are enhancing the spread of invasive alien plant species (IAPS) toward higher elevations, posing a new threat to mountains (Pyšek and Richardson 2010; Barni et al. 2012; Petitpierre et al. 2015; Alexander et al. 2016; Dullinger et al. 2017). Recent studies in the European Alps have shown that IAPS were able to spread upwards approximately twice as fast as natives (Dainese et al. 2014; Geppert et al. 2021). Therefore, understanding what drives the occurrence and the cover of IAPS along elevational gradients could provide new insights to prevent their spread in mountain plant communities. In this regard, the Mountain Invasion Research Network (MIREN) (Dietz et al. 2006; Pauchard et al. 2009; Alexander et al. 2011; Haider et al. 2022) has contributed in filling knowledge gaps on the distribution and ecology of IAPS in mountain regions worldwide (Iseli et al. 2023), and developed a standardized, long-term sampling protocol along mountain roads for monitoring changes in the elevational distribution, abundance, and composition of plant biodiversity (Haider et al. 2022). Indeed, upward shift of IAPS is being promoted by climate change as it was already observed in the monitoring sites of the MIREN network, where, over a 5–10 year period, the number of IAPS increased their upper range limit on average by ~ 16% per decade across regions (Iseli et al. 2023).

Roads are one of the main human-mediated drivers of IAPS dispersion and establishment in mountain ecosystems (Seipel et al. 2012). The dispersal of alien plants along roadsides is impacted by numerous factors, encompassing road characteristics, traffic density, and the nature and effectiveness of road maintenance practices (Vakhlamova et al. 2016). The linear configuration of road infrastructure creates a corridor-like environment that facilitates the natural expansion of IAPS over extended distances, as they encounter minimal obstacles to dispersal (Christen and Matlack 2009). Along elevational gradients, IAPS are typically first introduced and established at low elevations with greater propagule pressure and anthropogenic disturbances associated with settlements, agriculture, and industry (Lázaro-Lobo and Ervin 2019). Subsequently, IAPS can spread to higher elevations following road paths, as observed in several mountain regions (Alexander et al. 2011; McDougall et al. 2018; Haider et al. 2022; Barros et al. 2022). Despite many studies confirming these trends (McDougall et al. 2011; Alexander et al. 2016; Barros et al. 2022; Fuentes-Lillo et al. 2023), some mountain ranges are still considered resistant to invasion, especially at medium–high elevations (Iseli et al. 2023). The resistance to invasion may be contingent upon the capacity of native communities to tolerate it, or on local circumstances that are especially inhospitable to IAPS (Anderson et al. 2008). There is an assumed substantial risk of the expansion of IAPS from roadsides into natural areas, posing a threat to native plant communities. Both natural and human-mediated dispersal mechanisms, such as wind, animals, birds, turbulence from vehicles, and disturbances from maintenance activities, are potential contributors to the dissemination of alien plants from roadsides to adjacent natural habitats (Lavoie and Meunier 2012). Additionally, cultivated fields, construction sites, wastelands, or clear-cut areas in proximity to roadsides may serve as conduits for the spread of IAPS. Such disturbed habitats have the potential to act as stepping stones, facilitating the intrusion of these alien species into environmentally sensitive ecosystems (Follak et al. 2018). To sustain native biodiversity, plant species must exhibit resilience by either enduring unfavorable conditions or mechanical disturbances within plantations or possessing the capability to re-establish themselves following local extirpation (Heinrichs et al. 2018). However, little is known about the local ecological features of the native plant communities (Fried et al. 2019; Tordoni et al. 2020) that correlate with the occurrence and spread of IAPS in Mediterranean mountains. Native plant communities comprise species that are well adapted to local conditions and as such may serve as valuable indicators of ecological features and disturbances that may favour invasions. Disturbances that may create a disequilibrium in the distribution of resources potentially alter species composition through shifts in resource availability (Nogués-Bravo 2006; Romero-Díaz et al. 2017). This may, in turn, create conditions favoring the invasion of alien species (Amat et al. 2013).

Thus, recent and valuable tools that can be employed also to investigate the ecological features promoting biological invasions are the Ecological Indicator Values for Europe (EIVE) (Dengler et al. 2023) and the Disturbance Indicator Values (DI) (Midolo et al. 2023). Indicator values (e.g., Ellenberg indicator values) on climatic and edaphic niches of plant species have received considerable attention in ecological research (Arianoutsou et al. 2010b; Jiménez-Alfaro et al. 2021), whereas data on the optimal distribution of species along disturbance gradients have recently become available (Kermavnar and Kutnar 2024; Bricca et al. 2024). In particular, the indicators of light, temperature, and nitrogen (e.g., Landolt) were predictive and described ecological differences in communities, even in mountain environments (Varricchione et al. 2022; Ivanova and Zolotova 2023). Still, the new Disturbance Indicator Values for European species represent a valuable new instrument to improve knowledge on plant preferences in terms of disturbance types and intensity, particularly for certain types of disturbances historically common (e.g., grazing or mowing)(Dengler et al. 2014).

Plant invasion processes are particularly understudied in the Mediterranean mountains, and, consequently, the habitats and plant communities vulnerable to invasions in these environments are little known (Chytrý et al. 2008; Küzmič and Šilc 2017). In fact, mountains occupy more than 50% of land in many Mediterranean basin countries, seven of which are among the top 20 mountainous countries in the world (Blondel et al. 2010; Vogiatzakis 2012). The class ‘Mediterranean mountains’ encompasses low and medium mountains in the northern part of the Mediterranean basin and high mountains in the southern part, hosting unique and vulnerable plant diversity (Metzger et al. 2005). These mountains are also popular tourism destinations, having recreational, sporting, and aesthetic value. Over centuries, Mediterranean mountains have been greatly modified by humans, changing land use (e.g., agriculture, stockbreeding, or forestry) and impacting ecosystems in different ways. Therefore, anthropogenic factors have played a key role in shaping their environmental character (Cherif et al. 2020). The Mediterranean ecosystems face a higher susceptibility to the degradation of soil fertility, diminished water availability, and an elevated risk of forest fires as a consequence of global change compared to other European ecosystems (Schröter et al. 2005; Lindner et al. 2010; Peñuelas et al. 2017). Therefore, the likelihood of IAPS successfully adjusting to the Mediterranean basin's characteristics, particularly in the mountain areas, appears to be connected to their prior adaptation to the specific selective pressures of the Mediterranean biome. This includes their ability to conserve water and withstand challenges such as summer drought, fires, and low-nutrient soil (Cao Pinna et al. 2021). Moreover, the Mediterranean distribution of alien plants introduced from other continents depends on invasion pathways and histories, time since introduction, and climate match between native and invaded regions.

Nevertheless, while the IAPS occurrence in specific geographical regions of the Mediterranean basin has received research attention (Vilà and Muñoz 1999; Vilà et al. 2006; Arianoutsou et al. 2010a; Šilc et al. 2012; Camarda et al. 2016; Wagner et al. 2017; Lazzaro et al. 2020; Axmanová et al. 2021; Cao Pinna et al. 2021; Guarino et al. 2021; Spampinato et al. 2022), studies on IAPS specifically focusing Mediterranean mountain areas are still scanty. Understanding invasion processes and the role of road corridors for spreading alien plants in Mediterranean mountain ecosystems thus requires comparing the local native communities invaded with uninvaded ones, and their environmental conditions.

We hypothesized that Alien Plant Species (APS) richness is affected on a regional scale by elevation, which acts as a proxy for a range of climatic factors causing the decrease of alien species occurrence and cover along the elevation gradient. On a local scale, based on prior studies highlighting the increased richness of alien species in disturbed habitats, we expected to observe a higher occurrence of APS in edge habitats, particularly those adjacent to roadsides. Moreover, we expected to observe a higher occurrence and spread of APS in habitats characterized by resident native communities richer in disturbance-tolerant and light-demanding species.

In this study, we aim to investigate alien plant species occurrence and cover patterns in the Mediterranean mountain habitats found along road corridors in the Central Apennines (Italy), to pinpoint which local ecological and disturbance characteristics, inferred from native plant communities, correlate with APS occurrence and cover. In detail, we intend to answer to the following questions:

1. Which alien plant species are prevalent in the selected Mediterranean mountains?
2. What explains variation in alien plant species occurrence and cover?
3. Which ecological features and disturbance regimes of the native communities correlate with alien plant species occurrence and cover?

To develop effective management strategies and conservation efforts to mitigate the spread of invasive alien species and protect native biodiversity in Mediterranean mountain ecosystems, it is important to understand the invasion processes and ecological dynamics of these alien plant species. In this regard, this research may help to formulate policies aimed at minimizing human-mediated dispersal and growth of invasive alien plant species and preserving the unique and vulnerable plant diversity of Mediterranean mountain regions.

## Methods

### Study region and sampling design

The research was conducted in the Central Apennines mountain range in central Italy. The predominant geological composition of this area is limestone, and the mountain range experiences a climate that can be classified as warm-summer Mediterranean and temperate oceanic, as per the Köppen-Geiger classification system (Rita et al. 2023). The study area encompasses hilly and mountainous terrain with distinct vegetation types, including deciduous woodlands (*Quercus pubescens* Willd., *Quercus cerris* L., and *Fagus sylvatica* L. communities), *Pinus nigra* J.F.Arnold reforestation, *Lolium perenne* L. meadow, and *Bromus erectus* Huds grassland (Cutini, Blasi 2002; Biondi et al. 2014; Ciaschetti et al. 2015; Redowan 2015; Giarrizzo et al. 2017) (Supplementary Information Table S1).

**Fig. 1:**
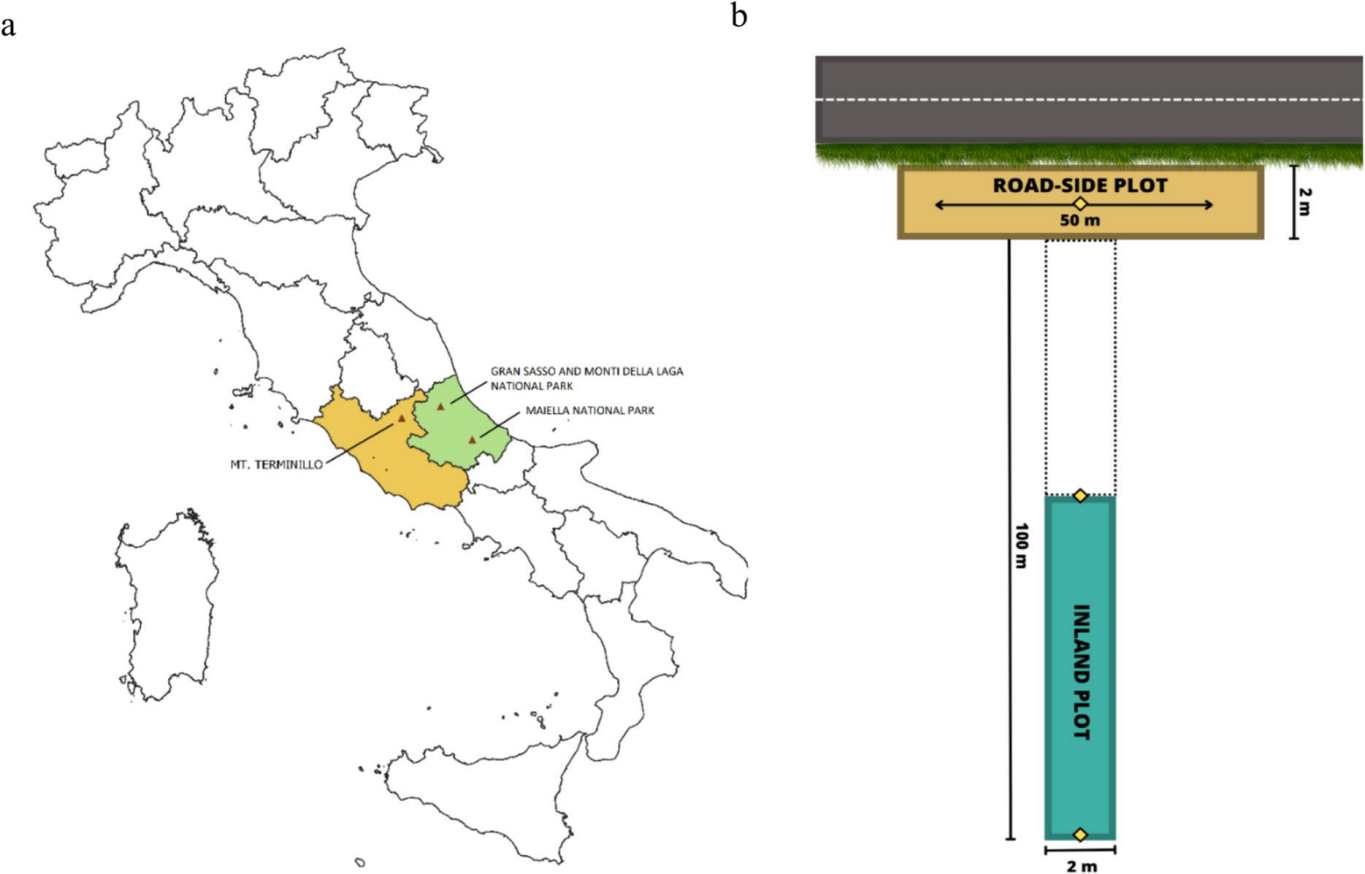
**a** Location of the three road transects sampled in Central Apennines (Italy). **b** In each road transect, sampling was conducted at 20 sites, each comprising two plots of 50 m × 2 m. The road-side plot is oriented parallel to the roadway, starting from the initial occurrence of vegetation along the road-side. The inland plot is positioned perpendicular to the road-side plot and is centrally located at a distance of 50 m

Vegetation sampling was performed during the summer of 2022, following the MIREN road survey protocol (Haider et al. 2022) consisting of vegetation surveys along three primary mountain roads. The three selected roads were located in the Gran Sasso and Monti della Laga National Park (from 595 to 2125 m a.s.l.), in the Maiella National Park (from 485 to 2065 m a.s.l.) and along Mt. Terminillo (from 420 to 1915 m a.s.l.) (Fig. 1a), and their lengths are reported as: (SS17bis) 21.5 km, (SP65), 10.5 km, and (SS4bis) 15 km, respectively. These three roads have comparable levels of traffic intensity and tourism during the year (Pignatti 1993; Piacentini et al. 2017; Poponi et al. 2022). The highest points of the road aligned with the most elevated spots on the mountain that were accessible to vehicles, such as places designated for refreshments and shelters. Conversely, the lowest points of the road were located in the valleys, where there was no remarkable change in elevation. Sampling locations were established by dividing the total elevation range of the roads into 19 elevational bands, (of ~ 100 m in elevation) giving a total of 20 sample sites. Sampling in each site was carried out on a transect. Each transect was designed in a T-shape configuration and consisted of two plots, each measuring 50 m in length and 2 m in width (Fig. 1b). The roadside plot was positioned in parallel with the road, while the inland plot was situated perpendicular to the center of the roadside plot, forming a 90° angle. The distance between the two plots was 50 m (Fig. 1b). In this study, although road peaks reached the alpine belt, we only considered sites up to approximately 1300–1400 m a.s.l, as we did not detect any APS much higher than 1200 m, also following previous studies that suggested similar elevational limit for many APS (Supplementary Information Fig. S1) (Alexander et al. 2016; Haider et al. 2018; Vorstenbosch et al. 2020). Therefore, for this study, we only considered plots located up to 1437 m above sea level (a.s.l.) in the Gran Sasso massif, 1415 m a.s.l. in the Maiella massif, and 1357 m a.s.l. at Mt. Terminillo.

For each plot, a few basic environmental variables and ancillary information were collected in the field, as elevation: in meters above sea level; obtained using a digital elevation model or Google Earth; at the same locations as geolocation is recorded, tree cover: percent (%) of plot covered by trees taller than 3 m. Bare ground: percent (%) of plot without vegetation (excluding rock and litter). Litter: percent (%) of the plot without vegetation but covered with dead and decaying plant material (such as leaves, bark, needles, and twigs). The cover of all vascular plant species was recorded in every plot. For each species, cover values (%) were estimated visually and later used as metric of cover in the analyses (eight percentage cover classes with 1 =  < 0.1%, 2 = 0.1–1%, 3 = 2–5%, 4 = 6–10%, 5 = 11–25%, 6 = 26–50%, 7 = 51–75%, 8 = 76–100%) (Haider et al. 2022). Tree cover was estimated considering projective foliage cover, which is defined as the proportion of the ground that is shaded by vegetation foliage when lit from directly above. Taxonomy and nomenclature followed (WFO 2023, Pignatti et al. 2017–2019; Bartolucci et al. 2018). Species were categorized as native (autochthonous species and species introduced before 1492, i.e., archaeophytes) and alien (species introduced after 1492, i.e., neophytes) (Galasso et al. 2018; Bartolucci et al. 2021, 2022; IPBES 2023). We recorded three categories of alien plant species (Pyšek et al. 2004), reported in Table 1. These categories are defined as follows (Galasso et al. 2018): (a) casual: alien plants that may thrive and even produce offspring occasionally outside cultivation, but that usually disappear because unable to form self-maintaining populations; their persistence relies on repeated introductions; (b) naturalized: alien plants that occur with self-maintaining populations without direct human intervention; (c) invasive: alien plants that occur with self-maintaining populations without direct human intervention, produce fertile offspring at considerable distances from the parent individuals, thus being able to spread over a large area.

**Table 1:** Alien Plant Species (APS) recorded in the study sites

Species	Family	Life form	Status	Origin area	Elevation range (m a.s.l.)	Road-side plots	Inland Plots
*Ailanthus altissima* (Mill.) Swingle	Simaroubaceae	P scap	Neophyte Invasive	East Asia	420–1190	25%	3%
*Robinia pseudoacacia* L	Fabaceae	P caesp	Neophyte Invasive	North America	677–1144	19%	3%
*Erigeron sumatrensis* Retz	Asteraceae	T scap	Neophyte Invasive	Central America	595–977	8%	6%
*Erigeron canadensis* L	Asteraceae	T scap	Neophyte Invasive	North America	419–1209	6%	6%
*Euphorbia prostrata* Aiton	Euphorbiaceae	T rept	Neophyte Invasive	North America	420–781	11%	0
*Isatis tinctoria* L	Brassicaceae	H bienn	Archeophyte Invasive	West Asia – North Africa	595–985	8%	0
*Senecio inaequidens* DC	Asteraceae	T scap	Neophyte Invasive	South Africa	900–985	6%	3%
*Aesculus hippocastanum* L	Sapindaceae	P scap	Neophyte Casual	West Asia	781–824	6%	0
*Artemisia verlotiorum* Lamotte	Asteraceae	H scap	Neophyte Invasive	East Asia	900–958	6%	0
*Hesperocyparis arizonica* (Greene) Bartel	Cupressaceae	P scap	Neophyte Naturalized	North America	533	6%	0
*Erigeron annuus* (L.) Desf	Asteraceae	T scap	Neophyte Invasive	North America	419–1056	3%	3%
*Bromus inermis* Leyss	Poaceae	H caesp	Neophyte Invasive	West Asia	985	3%	0
*Cedrus deodara* (Roxb. Ex. D.Don) G.Don	Pinaceae	P scap	Neophyte Naturalized	South Asia	533	3%	0
*Coreopsis lanceolata* L	Asteraceae	T scap	Neophyte Naturalized	North America	794	3%	0

Species names, family, life form (P scap: scapose Phanerophytes; P caesp: caespitose Phanerophytes; T scap: scapose Therophytes; T rept: reptant Therophytes; H bienn: biennial Hemicriptophytes; H scap: scapose Hemicriptophytes; H caesp: caespitose Hemicriptophytes; Raunkiær 1934), status in Italy (Galasso et al. 2018), origin area, observed elevational range (lower – upper limit in m a.s.l.), and species frequency in road-side plots and/or inland plot are reported

To achieve the objectives of our research, we used alien plant species occurrence and cover as dependent variables in statistical analyses, as we excluded them in the extraction of community ecological data, focusing only on native communities (Vilà et al. 2011; Fried et al. 2019).

### Ecological characteristics and level of disturbance of native communities

We used indicator values for native species to estimate the ecological characteristics and the level of disturbance of the invaded native communities. We calculated the Community-Weighted Means (CWM) for each sampled plot. Specifically, we computed the CWM by multiplying the trait value of each native species by its relative cover percentage within the plot, and then summing these values for all native species. Alien plant species were not included in these calculations.

To assess the ecological characteristics of native communities, we used the native species Ecological Indicator Values for Europe (EIVE) (Dengler et al. 2023), which indicate species preferences for temperature (EIVE T), nitrogen (EIVE N), soil reaction (EIVE R), soil moisture (EIVE M), and light (EIVE L). All these indicators range from 0 to 10, with lower values associated with sciaphilous or cold-tolerant species, and higher values associated with heliophilous and thermophilous species (using light and temperature as examples). Together, CWMs of these indicator values allow to describe well the ecological preferences of native communities and thus the local ecological conditions (Di Biase et al. 2023; Wrońska-Pilarek et al. 2023; Lepš and de Bello 2023), which might influence alien species occurrence and cover (Hansen et al. 2021).

In order to evaluate the ability of the native communities to withstand disturbance, we have assigned to each native species the corresponding Disturbance Indicator values (DI) (Midolo et al. 2023). These indicators refer to the species tolerance to: (a) the removal of the aboveground biomass by grazing, which ranged from 0 (no change in biomass) to 1 (complete loss of plant cover); (b) the soil disturbance, measured by the proportional increase in bare ground caused by furrowing or soil turning, ranging from 0 to 1; (c) the severity of disturbance in the herb layer by measuring the proportion of aboveground biomass removed, ranging from 0 to 1; (d) the frequency of disturbance events in the herb layer, expressed as the log_10_ mean inverse of return time in centuries; (e) the tolerance to mowing, measured by the log_10_ mean inverse of return time of mowing events in centuries.

### Data analysis

We opted for Random Forest (RF) as modelling algorithm. Random Forest is an ensemble learning method that constructs multiple decision trees to improve classification accuracy and robustness. Ecologically, it is particularly valuable for its ability to handle complex, non-linear relationships and interactions within large ecological datasets, reducing overfitting and providing reliable variable importance measures, aiding in the identification of key environmental predictors (Cutler et al. 2007). We employed two RF classification models to evaluate the variables that influenced (1) the occurrence of alien species, and (2) their cover. Both models used the CWMs for both EIVE and DI as continuous predictors, along with elevation, as well as the road transects (i.e., Gran Sasso, Maiella, Terminillo) and the position relative to the road (road-side/inland) as categorical predictors.

First, to explore the effects on the occurrence of alien species (defined as presence of at least one alien species in the community), we fitted a Random Forest Classification (RFC) model. Second, to explore the factors affecting the cover of the alien species, we fitted a RF Regression (RFR) model, testing the effect of the same predictors on the cover of alien plants (defined as the total cover of all alien species present in the plot) (Prasad et al. 2006; Cutler et al. 2007). In order to reduce multicollinearity and employ a non-redundant dataset, we conducted a Variance Inflation Factor (VIF) analysis of the set of continuous predictor variables using the function ‘vifstep’ of package ‘usdm’ (Naimi et al. 2014). A threshold of 5 was established for the VIF value (James et al. 2017). The VIF analysis retained the following continuous variables: Elevation, EIVE Moisture, EIVE Nitrogen, EIVE Reaction, EIVE Light, EIVE Temperature, Disturbance severity in the herb layer, Disturbance frequency in the herb layer, Mowing frequency, Grazing pressure, and Soil disturbance.

Both the RF models were subjected to a 5-time repeated tenfold cross-validation procedure. This was done to determine the optimal number of trees, with values tested at 50, 100, 500, 1000, 2000, and 5000. Additionally, the number of variables considered at each split (mtry) varied between 1 and 10. The selection of the ideal models was based on the Balanced Accuracy (BA) for RF classification and the Mean Absolute Error (MAE) for RF regression, respectively. In order to evaluate the goodness-of-fit of the models, we employed BA for the classification model and MAE and R^2^ for the regression model, respectively. The BA metric ranges from 0 to 1. It is calculated by taking the average of the sensitivity (i.e., the true positive rate) and the specificity (i.e., the true negative rate). Values of BA that are less than 0.5 can be interpreted as indicative of a random prediction (Velez et al. 2007). Variable importance was evaluated using the Mean Decrease in Accuracy (MDA) metric, expressed as a percentage, for the model on alien species occurrence (RF classification), and by measuring the rise in Mean Square Error (MSE), also expressed as a percentage, for the model on alien species cover (RF regression). Moreover, our analysis also investigated the effect shape of those variables which accounted for 10% of the total variable importance in each model. This was accomplished by generating partial dependence plots (PDP) for both the inland and road-side plots. All analyses were conducted using R version 4.2.1 (R Core Team 2022) using packages ‘caret’ (Kuhn 2008), ‘ggplot2’ (Wickham 2016), ‘randomForest’ (Breiman and Cutler 2001), ‘vegan’ (Oksanen et al. 2015) and ‘pdp’ (Greenwell 2017).

The RF models of native plant communities were tuned to include 5000 trees in both the classification and regression models. The value of mtry was adjusted to 4 for the classification model and 3 for the regression model. The goodness-of-fit of the final models yielded a BA value of 0.791 for the classification model, whilst the regression model exhibited an MAE of 4.33 and an R^2^ value of 0.258.

**Fig. 2:**
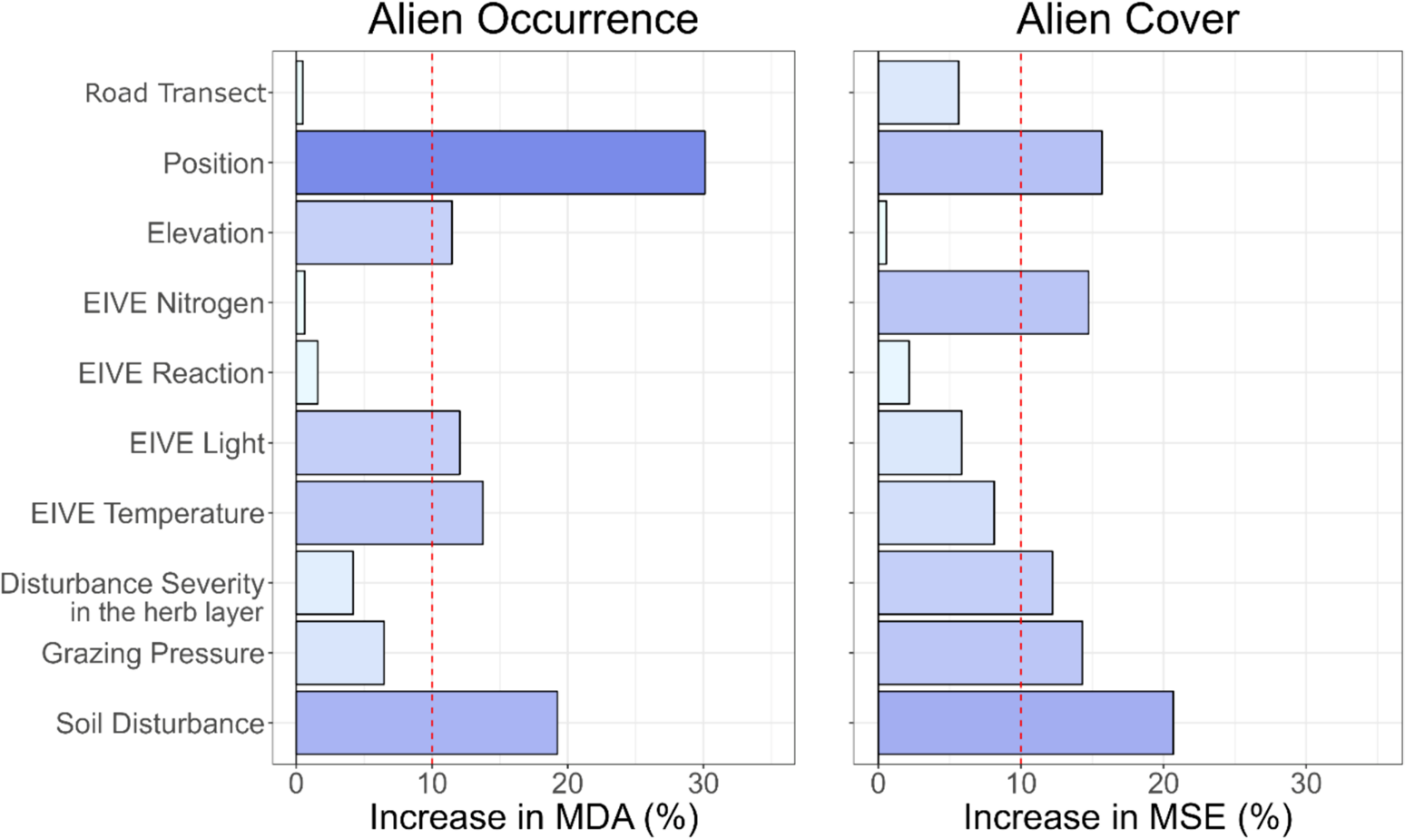
Variable Importance for Road Transect, Position relative to roads, Elevation, EIVE N, EIVE R, EIVE L, EIVE T, Disturbance severity for the herb layer, Grazing Pressure, and Soil Disturbance for both Alien Occurrence (**a**) and Alien Cover (**b**). For Alien Occurrence, the occurrence of at least one APS was used as the response variable and Mean Decrease in Accuracy (MDA) was used to assess variable importance, reported as a percentage. For Alien Cover, the cover of APS was used as the response variable and the increase in Mean Square Error (MSE) was used to assess variable importance, reported as a percentage

As the categorical variable 'Road Transect did not demonstrate any relevant importance in either model (Fig. 2), we set it to a reference value represented by the first massif in alphabetical order (i.e., Gran Sasso) when creating the Partial Dependence Plots. Instead, the categorical variable Position was of conspicuous relevance, especially for alien occurrence (Fig. 2). Thus, the PDPs were displayed by initially setting the value on the road-side and subsequently on inland. This enabled us to differentiate the interaction impact of the variable Position on each continuous variable of the two models and also evaluate a disparity in the probability of occurrence or cover based on the plot's position.

## Results

### General patterns of native and alien species

Overall, 655 different vascular plant species were recorded up to 1300–1400 m a.s.l. The most frequent native plant species (occurring in more than 50% of plots) were *Dactylis glomerata* L. *Brachypodium genuense* (DC.) Roem. & Schult.*, Clematis vitalba* L.*,* and *Sanguisorba minor* Scop*.* Species with high contribution to the total cover were *Fagus sylvatica, Brachypodium genuense, Fraxinus ornus* L.*, Quercus pubescens, Pinus nigra, Acer opalus* subsp*. obtusatum* (Waldst. & Kit. ex Willd.) Gams*, Clematis vitalba, Pteridium aquilinum* (L.) Kuhn*, Ostrya carpinifolia* Scop.*, Quercus cerris,* and *Dactylis glomerata* (Supplementary Information Table S3 and Table S4).

Across all plots, 14 species were alien plant species (Table 1). The Asteraceae family accounted for 43% of all APS found. As for the origin of the APS, 43% were from North America, followed by West Asia accounting for 21% of species. The upper distribution limit of APS was around 1200 m a.s.l. *Ailanthus altissima* (Mill.) Swingle, *Robinia pseudoacacia* L., and *Erigeron sumatrensis* Retz. were the most abundant APS in the sampled plots. APS were more common on road-side plots (plots number where APS occurred = 40) than on inland plots (n = 8). The elevation range with the highest number of APS was from about 420 m a.s.l. to 985 m a.s.l. Among the recorded APS, the prevalent life form was annual, but the trees *A. altissima* and *R. pseudoacacia* were the most frequent APS in the invaded sampled plots. In the road-side plots, also alien species contribute to the total cover (26%) such as *Ailanthus altissima, Robinia pseudoacacia, Juglans regia* L*., Pinus nigra,* and *Aesculus hippocastanum* L.

### Ecological features and disturbances favouring APS occurrence and cover

The Random Forest Classification (RFC) (Fig. 2a) showed that the most important variable that affected the probability of having at least one APS in the community was clearly the plot position relative to roads (road-side/inland), followed by two ecological/disturbance indicators exceeding 10% of Mean Decrease Accuracy: Soil Disturbance (Midolo et al. 2023), EIVE T (Temperature) and EIVE L (Light) (Dengler et al. 2023).

On the other hand, the Random Forest Regression (RFR) (Fig. 2b) showed that the most important variables that affected the cover of APS in the investigated plant communities were, in order of importance, Soil Disturbance, position relative to roads (road-side/inland), EIVE N (Nitrogen), Grazing Pressure, and Disturbance severity in the herb layer (Dengler et al. 2023; Midolo et al. 2023). All these variables exceeded 10% of Mean Square Error.

### Ecological features of native communities

**Fig. 3:**
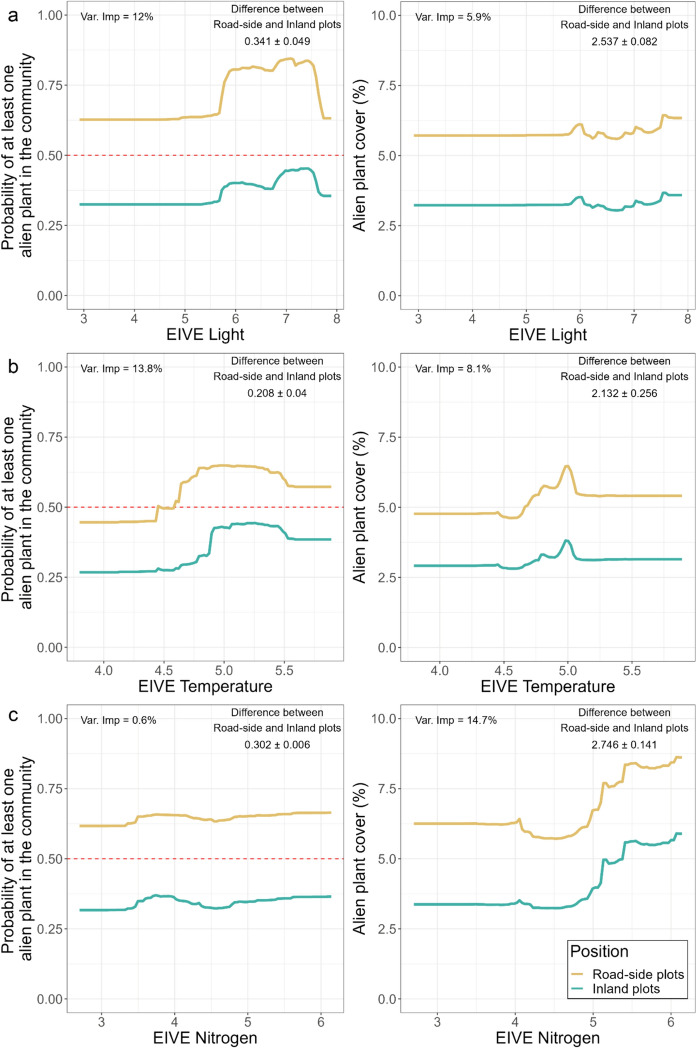
Partial Dependence Plots (PDP) for EIVE Light (**a**), EIVE Temperature (**b**) and EIVE Nitrogen (**c**) for the RF (Random Forest) model in the Central Apennine native communities. For RF Classification (left hand side), the probability of at least one APS existence in the road-side or inland plots relating EIVEs is shown by lines of different colours. The upper limit of the 50% probability of at least one APS occurrence is shown by the red dotted line. Similarly, for RF Regression (right hand side), the same is shown for APS cover. The mean ± standard deviation values between the inland and road-side plots, as well as variable importance (Var. Imp.) shown as Mean Decrease in Accuracy (MDA, RF classification) and the increase in mean squared error (MSE, RF regression), are displayed in the figures

The Community-Weighted Mean (CWM) for EIVE Light had a noticeable impact on the occurrence of APS in the native communities (Var. Imp. = 11.4%), although not as much on the abundance of APS, as shown by RFR (Var. Imp. 5.9%), according to Partial Dependence Plots (PDP) (Fig. 3a). In the RFC model, the effect size resulting from EIVE L revealed a mean difference in probability of at least one AP in the community of roughly 34% between inland and road-side plots. Communities with many light-demanding species (i.e., heliophile communities) demonstrated a higher likelihood of hosting at least one APS, which in road-side plots exceeds 75% probability of occurrence when the EIVE Light CWM was in the range of 5.5–7.5. As already mentioned, in RFR model, the Community Weight Mean for EIVE L showed a low effect on the cover of APS, showing only a mean difference in cover between road-side and inland plots of about 2.5%.

Regarding the PDP for EIVE T (Fig. 3b), it can be shown that the variable importance on RFC is almost twice as high as on RFR (Var. Imp. 15.5 vs. 8.1%, respectively). Particularly, it was found that even in road-side plots, more cold-adapted communities with CWMs for EIVE T < 4.5 have a low chance of hosting at least one APS (below 50%). On the other hand, the chance of hosting at least one APS increases for thermophilic communities with values between 4.5 and 5.5; this probability plateaued at constant values beyond 5.5. On average, the difference in probability between the road-side and inland plots of hosting at least one APS is narrower with regard to EIVE T. Although the effect of EIVE T was less pronounced on the cover of APS, the PDP showed an increase in cover between the values of 4.5 and 5, with a subsequent levelling off. The average difference in coverage between road-side and inland plots was about 2%.

Nitrogen affinity in the community (EIVE N) had a large effect on the cover of APS (Var. Imp. = 14.7%), in contrast to the CWM for light affinity, but no influence on the likelihood of harboring at least one APS (Var. Imp. = 0.5%; Fig. 3c). Particularly, there was a minor decline in APS cover between the values of 4 and 5 of the CWMs for nitrogen, notably in the road-side plots. However, there was a discernible increase in APS cover above 5. On average, between outer and inner plots, there was a difference in APS cover of about 3%.

### Disturbance regimes of native communities

**Fig. 4:**
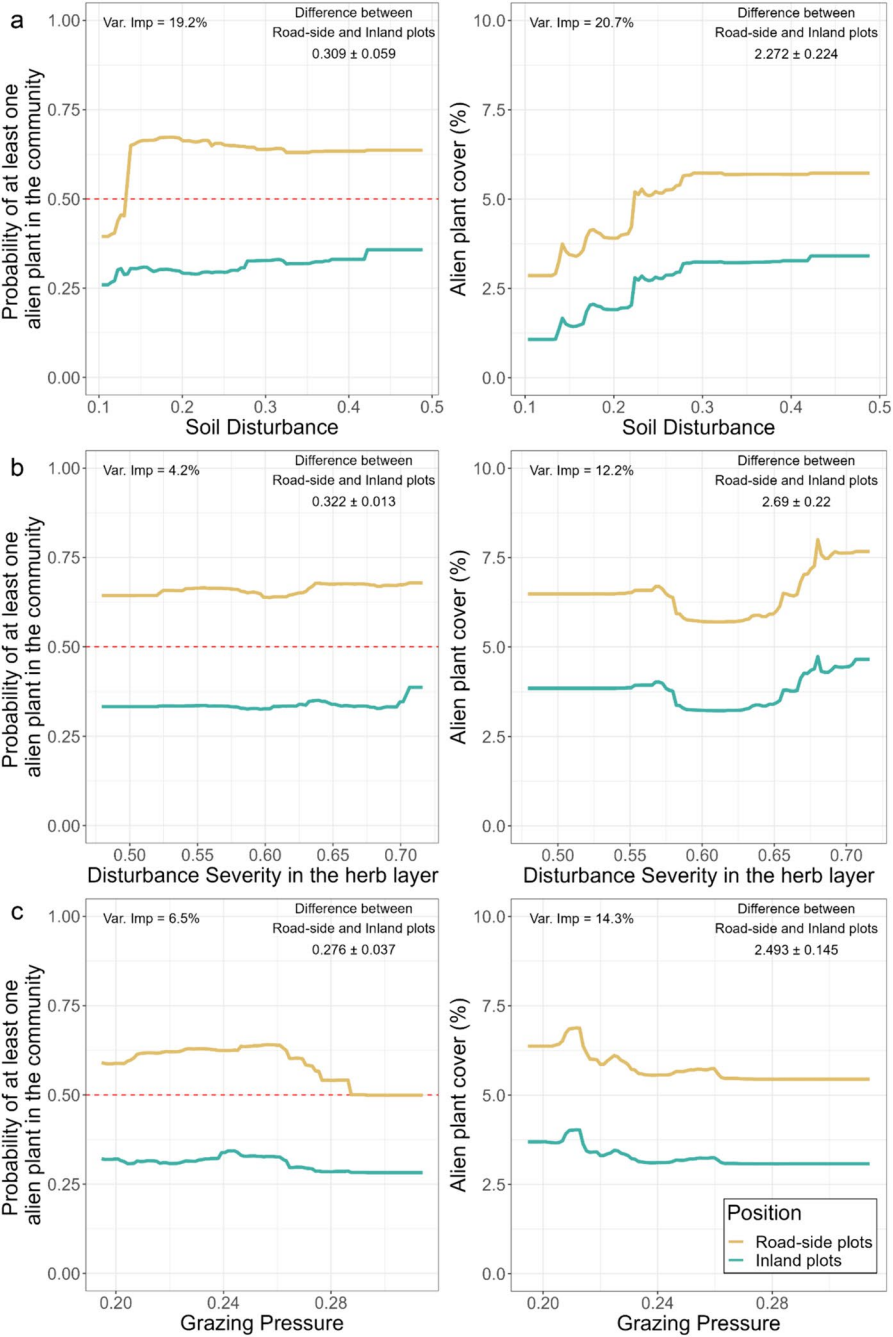
Partial Dependence Plots (PDP) for Soil Disturbance (**a**), Disturbance severity in herb layer (**b**), and Grazing Pressure (**c**) for the RF (Random Forest) model in the Central Apennine native communities. For RF Classification (left hand side), the probability of at least one APS existence in the road-side or inland plots relating DIs is shown by lines of different colours. The upper limit of the 50% probability of at least one APS occurrence is shown by the red dotted line. Similarly, for RF Regression (right hand side), the same is shown for APS cover. The mean ± standard deviation values between the inland and road-side plots, as well as variable importance (Var. Imp.) shown as Mean Decrease in Accuracy (MDA, RF classification) and the increase in mean squared error (MSE, RF regression), are displayed in the figures

CWMs for Soil Disturbance showed a considerable effect on both the APS occurrence in the native community and their cover, with variable importance of around 20% in both cases (Fig. 4a). In particular, the PDP for alien occurrence showed that, in the inland plots, Soil Disturbance had no relevant effect, whereas in the road-side plots there was a clear threshold value of about 0.15, above which the probability of having at least one APS in the native community remained high and constant around 67%. A strong difference between road-side and inland plots was consequently evident, with the average difference in the probability of alien occurrence in communities being around 31% in road-side plots compared to inland ones. In contrast, the model for alien cover showed that Soil Disturbance influenced APS cover in both inland and road-side plots, with an almost linear increase between the values of 0.1 and 0.3, and a subsequent levelling off. On average, there was a difference in APS cover between road-side and inland plots of about 2%.

Disturbance Severity (in the herb layer) (Fig. 4b) had no effect on the occurrence of alien species in the communities (RFC Var. Imp. = 0%, Fig. 4), but a notable effect on APS cover (RFR Var. Imp. = 12.2%). In particular, there was no effect of Disturbance Severity for values below 0.55. Between 0.55 and 0.65, on the other hand, there was a slight decrease in the APS cover values, an effect that was more pronounced in the road-side plots. Above a value of 0.65, in contrast, a rather strong increase was shown in the APS cover values, which was again more pronounced in the road-side plots. In general, between the roadside and inland plots, there was an average difference in the coverage of the APS of about 3%.

Finally, CWM for Grazing Pressure (Fig. 4d) also showed a significant effect on alien cover (Var. Imp. = 14.3%) but little significance with regard to alien occurrence (Var. Imp. = 3.9%). The cover of the APS tended to decrease between values of 0.21 and around 0.27, with a subsequent levelling off above the value of 0.28, even though the effect was not overly pronounced and there was a narrower mean difference in the cover of road-side plots and inland plots (around 2.5%), despite some fluctuations with slight increase of AP cover around Grazing Pressure values of around 0.21.

## Discussion

### General patterns of alien plant species

Overall, we found 14 alien plants occurring along roads in our Mediterranean mountain region. The total number of alien species in our study area is in line with what was found in other MIREN mountain sites as in Austria (14) and Norway (16), while lower than the total found in Switzerland (32) and the Czech Republic (25). The most abundant woody APS recorded in the sampled area were *Ailanthus altissima* and *Robinia pseudoacacia*. These two alien trees are very common in Europe (Campagnaro et al. 2018) and in the Mediterranean basin (Lazzaro et al. 2020; Guarino et al. 2021; Kalusová et al. 2023). Their upper elevation limit in our study area is comparable with those recorded in other areas of the Mediterranean basin. Indeed, we found that *Ailanthus altissima* reaches the highest elevation at 1190 m a.s.l. and this range threshold is consistent with what was recorded in Croatia (Vitasović-Kosić et al. 2020) and Spain (Lapiedra et al. 2015). The maximum elevation recorded for *Robinia pseudoacacia* in our study area is 1144 m a.s.l., as was also observed in Southern Italy (Saulino et al. 2023) and in Croatia (Vitasović-Kosić et al. 2020). Among herbaceous alien plants, *Erigeron canadensis* and *E. sumatrensis* were the most common ones. In our study area, they did not exceed the elevation of 1209 m a.s.l, even if they were recorded at higher elevations in other mountains of the Mediterranean basin, reaching 1831 m a.s.l. in Croatia while in Slovenia they were found up to 902 m a.s.l (Nikolić et al. 2013; de Groot et al. 2016; Vitasović-Kosić et al. 2020). Another very common alien species in Mediterranean mountains is *Senecio inaequidens*, which we found up to 985 m a.s.l. in our study area. This threshold is consistent with what was recorded in Spain (Castells et al. 2014). Thus, the distribution of the most common alien plants along our elevation gradient aligns well with patterns recorded throughout the mountains of the Mediterranean region.

We found that in our Mediterranean mountains, as is the case in other elevation gradients of the MIREN network, APS were most frequent and had higher cover at the road-side plots in comparison with inland plots (Lembrechts et al. 2014; Haider et al. 2022). Proximity to roads was by far the most important factor for determining alien species occurrence, while it was not as important for determining their cover. The road-side plots are often characterized by a "mosaic" of natural and anthropogenic habitats: this is due to the elongated shape of the plot parallel to the road and the influence of the latter. Indeed, road-side habitats are often shaped by extensive anthropogenic habitat modifications and disturbances associated with the maintenance of road infrastructures, which typically favour alien plants (Lázaro-Lobo and Ervin 2019). These factors seem to be just as important for promoting alien plant invasions in the Mediterranean region, confirming that also in this area roads represent important dispersal corridors for further invasions into mountain areas. This was an expected result for us, as the study area is very frequented by tourists throughout the year. In fact, it is possible to visit the mountains and the parks during summer for hiking and outdoor activities on foot, by mountain bike or on horseback, while in winter there are ski resorts that give the opportunity to practice downhill skiing, cross-country skiing, and snowboarding.

Moreover, APS occurred more frequently in the hilly landscape where *Quercus cerris* and/or *Q. pubescens* woods were fragmented and replaced by tall herb fringe communities, ruderal vegetation, and cultivated fields (Supplementary Information Table S1). This is consistent with the findings reported by Viciani et al. (2020) who recorded the highest number of alien species in annual and perennial ruderal herbaceous hilly vegetation of Italy. Similarly, Barni et al. 2012 found most of the invasive plant species at low and medium elevations in western Alps, below 1430 m a.s.l., and they were usually ruderal or ruderal-competitive species with high ecological plasticity. Currently, mountain beech forests, heathlands, and herbaceous alpine habitats (e.g., alpine grasslands, snow beds, screes and rocky slopes) represent a barrier to APS invasion in the study area. This is consistent with patterns recorded in the Alps, where montane spruce forest is considered an effective barrier against the establishment of alien species (Siniscalco et al. 2011; Barni et al. 2012; Vorstenbosch et al. 2020). Indeed, in central Apennine, beech forests, due to high shading and thick litter, represent a hostile environment to the growth of species in the herbaceous and shrub layer (Pauchard and Alaback 2004; Averett et al. 2016). At the same time, also the severe climate of the high Mediterranean mountains constitutes a barrier to the rooting and spread of thermophilous species (Morales-Molino et al. 2021; Varricchione et al. 2021; Aurelle et al. 2022) and this also works for the current set of alien plants present in the Central Apennines, that barely grow beyond 1200 m a.s.l. This confirmed the hypothesis that climate and competition barriers preclude the establishment and spread for new species arriving in high mountain habitats, where high mortality of seedlings and slow growth rates occur (Alexander et al. 2016). Specifically, climatic conditions could represent a barrier both along the elevation gradient and the road-side and inland plots gradient, while the competition barrier is mostly relevant to the horizontal gradient (inland plots). This happens in many other mountain groups on the planet (Alexander et al. 2011; Nikolić et al. 2013; Castells et al. 2014; Lapiedra et al. 2015; McDougall et al. 2018; Vitasović-Kosić et al. 2020), except for tropical and sub-tropical mountains greatly frequented by hikers where alien plants were found at higher elevations (Hemp 2008; Pauchard et al. 2009; Seipel et al. 2012; Alexander et al. 2016; Haider et al. 2022; Iseli et al. 2023).

### Ecological features for native communities

The indirect analysis of the ecological characteristics and disturbance regime of native plant communities showed that the probability of alien occurrence in the native communities increased mainly in communities adapted to high light availability and warm conditions (higher values of EIVE T and EIVE L). These conditions mainly occur when forest cover is interrupted, and vegetation is cut or burned. As already described in the literature, APS take advantage of forest cutting and wildfire disturbance to colonize new habitats (Maringer et al. 2012; González-Moreno et al. 2013; Averett et al. 2016; Chiuffo et al. 2018; Saulino et al. 2023). Indeed we found that in thermophilic communities (EIVE T values between 4.5 and 5.5) the chance of harboring alien species increased both in road-side and inland plots. This phenomenon did not occur only in semi-dry perennial calcareous grassland at low elevations but also in *Pinus nigra* plantation communities at higher elevations (822–1069 m a.s.l.). This means that the replacement of the potential vegetation of the temperate deciduous forest with pine plantations created a favorable environment for the establishment of alien species (Heinrichs et al. 2018).

In parallel, we also found that higher alien cover was associated with communities adapted to high nitrogen availability (higher EIVE N values), with slightly higher values in road-side plots, as it is known, roads indeed cause altered soil properties, including higher nutrient levels, changes in soil pH, increased drainage and more extreme microclimatic conditions (Forman and Alexander 1998; Alexander et al. 2016; Ratier Backes et al. 2021; Fuentes-Lillo et al. 2023).

Alien species often exhibit traits that give them strategic advantages over native plants in utilizing nutrients, particularly nitrogen and phosphorus (Lee et al. 2017). Moreover, even non-N-fixing invasive species are associated with increases in soil N pools and fluxes (Castro-Díez et al. 2014), and the enrichment of soil nutrients can facilitate the growth of native ruderal and nitrophilic species, as well as secondary invasions by non-native species (Gioria and Osborne 2014; Gioria et al. 2023).

In particular, native species indicators of high N availability include *Acer pseudoplatanus* L.*, Juglans regia, Hordeum murinum* L.*, Lolium perenne, Rubus idaeus* L.*, Clematis vitalba* (Supplementary Information Table S2 and Table S3). For instance, *A. pseudoplatanus* and shrubby species such as *R. idaeus* typically grow in large forest clearings or at the edges of beech woods as well as along trails (paths/corridors) frequented by humans and animals with nutrient accumulation (Simon et al. 2014; Li et al. 2015). The other N-adapted species are typical of the clearings occurring in lower bioclimatic belts, e.g. in *Quercus* woods fringe and/or on the edges of the roads, where ruderal and generalist species prevail (Lembrechts et al. 2014).

### Disturbance regimes for native communities

Moreover, medium–high levels of Soil Disturbance, Grazing Pressure, and Disturbance Severity in the herb layer were linked to higher APS cover. In particular, Soil Disturbance was the most important factor explaining the cover of alien species, even more than proximity to the road. Indeed, these Disturbance Indicators had the strongest effect in plant communities next to roads, as road-side plots are subjected to periodic road-side maintenance, trampling for the parking of cars, passage of people and domestic/bred animals, and littering (Forman and Alexander 1998). These conditions may facilitate the colonization of alien plants by suppressing the growth or removing stands of native species (Trombulak and Frissell 2000; Bacaro et al. 2015). In general, disturbance reduces total plant biomass, facilitating alien plants because of reduced competition with natives for light and resources (Lembrechts et al. 2016; Corcos et al. 2020). Moreover, along road-sides an increased human-mediated propagule pressure increases the probability of plant invasions (Simberloff 2009), and, when propagule pressure is coupled with climate warming, the spread of alien plants is greater and reaches higher elevations (Carboni et al. 2018).

Species associated with higher values of Soil Disturbance (furrowing or soil turning) include *Avena fatua* L.*, Vicia sativa* L.*, Convolvulus arvensis* L*,* native plant species in the Mediterranean basin that grow in ruderal vegetation in soils recurrently tilled (Maccherini 2006; Porqueddu et al. 2017) identifying annual herb-dominated habitats (Chytrý et al. 2020). Indeed, one of these species, *Convolvulus arvensis,* is considered an alien plant species in the Central Andes of Chile and Argentina and it was associated with the disturbance caused by tourism (informal trails) and cattle (Fuentes-Lillo et al. 2023). Soil disturbance was among the most important predictors of the probability of alien species occurrence, especially it supports the establishment of annual/herbaceous species (*Senecio inaequidens*, *Erigeron sumatrensis* and *E. canadensis*) and seedlings of alien trees. Soil disturbance generating gaps in plant cover also allows the establishment of generalist species, i.e. *Arabis hirsuta* (L.) Scop*., Galium aparine* L. and *Poa pratensis* L., as it was recorded in Geppert et al. (2021), only a limited subset of native species was able to colonize the disturbed soil. The removal of resident vegetation and the creation of bare ground through soil disturbance indeed facilitated the establishment of non-native species, aligning with previous findings (Lembrechts et al. 2016). This reaffirms the importance of an invading species having access to vital resources such as light, nutrients, and water. Success in invading a community is more likely when the APS faces less intense competition for these resources from native species (Davis et al. 2000). Many alien species exhibit traits linked to faster growth rates and efficient resource acquisition (Van Kleunen et al. 2010), likely enabling them to exploit the resources made available by soil disturbance at the expense of native species (Haider et al. 2018; McDougall et al. 2018) (Supplementary Information Table S2 and S3).

Regarding Disturbance Severity values, that was measured considering the proportion of aboveground biomass (herb layer) killed by disturbance (Midolo et al. 2023), a notable effect on alien plants cover was recorded. Species of road-side native communities with high values for that ecological index are *Avena fatua, Aegilops geniculata* Roth and *Carthamus lanatus* L. which are typical of the annual anthropogenic grassland of areas subjected to frequent disturbance (cutting, trampling, soil re-arrangement) (Chytrý et al. 2020).

In our study case, alien plants cover tends to a slight increase in plant communities with medium values of Grazing Pressure. As reported by Keeley et al. (2003), grazing pressure may vary seasonally and as a function of livestock type, stocking density, timing, and duration. The impact of grazing on native species richness and community invasibility is variable and likely to be complicated by grazing severity and timing, individual species responses, and abiotic factors such as soil characteristics and light availability. The native *Lolium perenne, Festuca circummediterranea* Patzke, *Trifolium scabrum* L. are the species with the highest indicator values for grazing pressure and are typical of the Mediterranean hill pastures where the grazing pressure is at a medium–low level (Caballero et al. 2009).

In general, therefore, the native plant communities farthest from the road are poorly affected by the occurrence of APS, as their floristic composition, their structure and ecological characteristics limit the establishment of these species. In fact, APS were poorly represented in plant communities away from the road compared to those at the road verges. This could change, however, because of climate change, which induces in the Mediterranean mountains an increase in average temperature and an increase in summer dryness (Cutini et al. 2021; Aurelle et al. 2022). This process could lead over time to an expansion in the hilly and submontane belts of more heliophilous and thermophilic communities, which result from our study more prone to colonization by APS (Iseli et al. 2023).

## Conclusions

Mediterranean mountains are peculiar environments that host unique plant diversity and are particularly fragile in the face of ongoing global changes. Plant invasions represent a new threat in these systems which is not yet well understood. The local environmental characteristics and disturbance regimes in native plant communities along mountain roads may play a crucial role in determining whether alien plant invasions are successful and able to spread into mountains. Our work provided new insights into alien plant invasion patterns in the mountain vegetation of the central Apennines (Italy), in the Mediterranean region, and, using indicator values of native plant communities, identifying which local ecological characteristics and disturbance factors affect alien plant occurrence and cover.

The results showed that, although alien species are present, their occurrence is mostly limited to areas close to the road and does not exceed 1200 m a.s.l. of elevation, showing a good degree of ecological resistance of inland native communities. In the medium- and long-term period, global warming may potentially challenge the current resistance and could potentially facilitate the spread of aliens to inland areas. The medium to high degree of thermophilia and heliophilia within plant communities may facilitate the establishment of alien species.

We also confirmed that the disturbance affecting the soil and the biomass of the native community promotes the establishment and the spread of aliens, indicating that the management of road edges must be improved to reduce gaps in native vegetation.

Our research provided the first baseline for monitoring alien plant occurrence in Mediterranean mountains over time according to the sampling protocol of the MIREN project. This will allow a continuous update on the invasion processes in place and will allow to make comparisons with other mountain regions monitored with the same sampling protocol in other mountains of the planet, contributing to providing useful data for the management of alien species in mountain areas at national and European levels.

## Supplementary Information

Below is the link to the electronic supplementary material.

Supplementary file1 (DOCX 2443 kb)

## Data Availability

Data analysed during the current study are available from the corresponding author upon reasonable request.
