# Supplementary material for: Ecological features facilitating spread of alien plants along Mediterranean mountain roads
Source: Biol Invasions. 2024 Aug 8;26(11):3879–99. doi: 10.1007/s10530-024-03418-y (PMC11420372; doi:10.1007/s10530-024-03418-y)
Supplement: Supplementary file 1 — Supplementary file1 (DOCX 2443 kb) [file 10530_2024_3418_MOESM1_ESM.docx]

**Supplementary Information (SI)**

**ECOLOGICAL FEATURES FACILITATING SPREAD OF ALIEN PLANTS ALONG MEDITERRANEAN**

**MOUNTAIN ROADS**

**Lucia Antonietta Santoianni^1^, Greta La Bella^2^, Marta Carboni^2,4^, Sylvia Haider^3^, Michele Innangi^1^, Marco Varricchione^1,4^, Angela Stanisci^1,4^**

^1^EnviXLab, Department of Biosciences and Territory, University of Molise, Termoli and Pesche, Italy

^2^Department of Science, Roma Tre University, Rome, Italy

^3^Institute of Ecology, Leuphana University of Lüneburg, Lüneburg, Germany

^4^National Biodiversity Future Center (NBFC), Palermo (PA) 90133, Italy

We carried out a cluster analysis for classifying the vegetation of the study area. We analysed a matrix of 70 plots × 291 species using a K-means cluster analysis in R environment (R Core Team 2022). The 70 plots were 36 road-side plots and 34 inland plots (2 internal plots were not used because were not accessible for sampling). In the cluster analysis we only included species with a total coverage higher than 1%. In order to determine the best suitable number of clusters, we employed the ‘fviz_nbclust’ function from the ‘factoextra’ package (Kassambara and Mundt 2020), utilising the ‘silhouette’ approach. The analysis revealed that the optimal outcome was achieved with 9 clusters and plots were thus classified in the 9 cluster groups. For each group the diagnostic species were identified using the ‘Indicator Species’ function in the PAST software (Hammer et al. 2001), and compared with the diagnostic, dominant and constant species of EUNIS habitats (Chytrý et al. 2020)(Table 1).

**Table S1** The table shows the vegetation types occurring in the sampled plots, according to EUNIS habitat classification (Chytrý et al. 2020). EUNIS habitat codes, the full habitat name, the diagnostic, dominant, and constant species detected in the sampled plots, the total number of plots referred to each EUNIS habitat, and the cluster number are reported**.**

| **EUNIS HABITAT CODE** | **EUNIS HABITAT NAME** | **DIAGNOSTIC, DOMINANT**  **AND CONSTANT TAXA** | **N. PLOTS** | **CLUSTER NUMBER** |
| --- | --- | --- | --- | --- |
| **R1A/R51/V34** | Semi-dry perennial calcareous grassland (meadow steppe) / Thermophilous forest fringe of base-rich soils / Trampled xeric grassland with annuals | *Anthoxanthum odoratum* L.*, Anthyllis vulneraria* L.*, Acer campestre* L.*, Rubus ulmifolius* Schott*, Lolium perenne* L*., Plantago lanceolata* L.*, Convolvulus arvensis* L. | 46 | 7 |
| **R14/R18** | Perennial rocky grassland of the Italian Peninsula / Perennial rocky calcareous grassland of subatlantic-submediterranean Europe | *Bromus erectus* Huds.*, Festuca inops* De Not.*, Globularia bisnagarica,* L., *Helianthemum canum* (L.) Hornem., *Teucrium chamaedrys* L. | 4 | 9 |
| **R54** | Pteridium aquilinum vegetation | *Pteridium aquilinum* (L.) Kuhn, *Achillea millefolium* L.*, Holcus lanatus* L. | 2 | 4 |
| **T17** | Fagus forest on non-acid soils | *Fagus sylvatica* L. *,Cardamine bulbifera* Crantz*, Prenanthes purpurea* L. | 9 | 1/2/3 |
| **T19** | Temperate and submediterranean thermophilous deciduous forest | *Quercus cerris* L.*, Acer opalus subsp. obtusatum* (Waldst. & Kit. Ex Willd.) Gams*, Sorbus torminalis* (L.) Crantz*, Carex flacca* Schreb. | 3 | 6 |
| **T1A** | Mediterranean thermophilous deciduous forest | *Quercus pubescens* Willd.*, Hippocrepis emerus subsp. emerus* (L.) Lassen*, Cornus sanguinea* L. | 4 | 8 |
| **T3M/T19** | Coniferous plantation of non site-native trees / Temperate and submediterranean thermophilous deciduous forest | *Pinus nigra* J.F.Arnold*, Fraxinus ornus* L.*, Prunus spinosa* L. | 2 | 5 |

***Table S2*** *The table displays for the pair plots (roadside/inland) the average elevation with standard deviation* *and the EUNIS habitat codes* *(Chytrý et al. 2020) corresponding to the vegetation types (see Table 1 for EUNIS habitat description).*

|  | **PLOT** | **ELEVATION ± SD**  **(m a.s.l.)** | **ROAD-SIDE PLOT** | **INLAND PLOT** |
| --- | --- | --- | --- | --- |
| GRAN SASSO | 1 | 595±0 | R1A/R51/V34 | R1A/R51/V34 |
|  | 2 | 687±14 | R1A/R51/V34 | R1A/R51/V34 |
|  | 3 | 724±0 | R1A/R51/V34 | R1A/R51/V34 |
|  | 4 | 808±20 | R1A/R51/V34 | T3M/T19 |
|  | 5 | 906±8 | R1A/R51/V34 | R14/R18 |
|  | 6 | 989.5±6 | R1A/R51/V34 | R14/R18 |
|  | 7 | 1065.5±5 | R1A/R51/V34 | T3M/T19 |
|  | 8 | 1137±10 | R1A/R51/V34 | R1A/R51/V34 |
|  | 9 | 1190±0 | R1A/R51/V34 | R1A/R51/V34 |
|  | 10 | 1280±0 | T17 | T17 |
|  | 11 | 1361.5±6 | R1A/R51/V34 | R1A/R51/V34 |
|  | 12 | 1429.5±11 | R1A/R51/V34 | R1A/R51/V34 |
| MAIELLA | 1 | 492.5±11 | T1A | R1A/R51/V34 |
|  | 2 | 533±0 | R1A/R51/V34 | T1A |
|  | 3 | 628±0 | R1A/R51/V34 | R54 |
|  | 4 | 712.5±4 | R14/R18 | R54 |
|  | 5 | 785.5±6 | R1A/R51/V34 | R1A/R51/V34 |
|  | 6 | 842.5±8 | R1A/R51/V34 | R1A/R51/V34 |
|  | 7 | 965.5±16 | R1A/R51/V34 | R1A/R51/V34 |
|  | 8 | 1066±14 | R1A/R51/V34 | R1A/R51/V34 |
|  | 9 | 1132.5±12 | R1A/R51/V34 | R1A/R51/V34 |
|  | 10 | 1213±13 | T17 | T17 |
|  | 11 | 1277.5±4 | T17 | T17 |
|  | 12 | 1386±41 | T17 | T17 |
| TERMINILLO | 1 | 419.5±1 | R1A/R51/V34 | R1A/R51/V34 |
|  | 2 | 489±31 | R1A/R51/V34 | R1A/R51/V34 |
|  | 3 | 555.5±1 | R1A/R51/V34 | R1A/R51/V34 |
|  | 4 | 672±0 | T1A | - |
|  | 5 | 774±10 | R1A/R51/V34 | R1A/R51/V34 |
|  | 6 | 817±11 | R1A/R51/V34 | T19 |
|  | 7 | 899±25 | R1A/R51/V34 | T1A |
|  | 8 | 1038±0 | R1A/R51/V34 | - |
|  | 9 | 1112±37 | T17 | R1A/R51/V34 |
|  | 10 | 1119±1 | R1A/R51/V34 | R1A/R51/V34 |
|  | 11 | 1205±6 | R1A/R51/V34 | T19 |
|  | 12 | 1339±24 | R14/R18 | T19 |

***Table S3*** *List of 655 species recorded in the study area, with family and status (native, archaeophyte, neophyte). The nomenclature follows* World Flora Online (2023) (Kindt 2020) *and for alien plant species follows* Galasso et al. (2018), Bartolucci et al. (2021, 2022), IPBES (2023).

| **SPECIES** | **FAMILY** | **STATUS** |
| --- | --- | --- |
| *Abies alba* Mill. | Pinaceae | Native |
| *Acer campestre* L. | Sapindaceae | Native |
| *Acer cappadocicum* subsp. *lobelii* (Ten.) A.E.Murray | Sapindaceae | Native |
| *Acer monspessulanum* L. | Sapindaceae | Native |
| *Acer opalus* subsp. *obtusatum* (Waldst. & Kit. Ex Willd.) Gams | Sapindaceae | Native |
| *Acer platanoides* L. | Sapindaceae | Native |
| *Acer pseudoplatanus* L. | Sapindaceae | Native |
| *Achillea collina* (Becker ex Rchb.f.) Heimerl | Asteraceae | Native |
| *Achillea millefolium* L. | Asteraceae | Native |
| *Achillea setacea* Waldst. & Kit. | Asteraceae | Native |
| *Adenostyles australis* (Ten.) Iamonico & Pignatti | Asteraceae | Native |
| *Adonis flammea* Jacq. | Ranunculaceae | Native |
| *Aegilops geniculata* Roth | Poaceae | Native |
| *Aegonychon purpurocaeruleum* (L.) Holub | Boraginaceae | Native |
| *Aesculus hippocastanum* L. | Sapindaceae | Neophyte |
| *Aethionema saxatile* (L.) W.T.Aiton | Brassicaceae | Native |
| *Agrimonia eupatoria* L. | Rosaceae | Native |
| *Agrostis canina* L. | Poaceae | Native |
| *Agrostis stolonifera* L. | Poaceae | Native |
| *Ailanthus altissima* (Mill.) Swingle | Simaroubaceae | Neophyte |
| *Aira caryophyllea* L. | Poaceae | Native |
| *Ajuga reptans* L. | Lamiaceae | Native |
| *Alliaria petiolata* (M.Bieb.) Cavara & Grande | Brassicaceae | Native |
| *Allium scorodoprasum* L. | Amaryllidaceae | Native |
| *Allium sphaerocephalon* L. | Amaryllidaceae | Native |
| *Allium vineale* L. | Amaryllidaceae | Native |
| *Alnus glutinosa* (L.) Gaertn. | Betulaceae | Native |
| *Alopecurus pratensis* L. | Poaceae | Native |
| *Alyssum alyssoides* L. | Brassicaceae | Native |
| *Amelanchier ovalis* Medik. | Rosaceae | Native |
| *Anacamptis morio* (L.) R.M.Bateman, Pridgeon & M.W.Chase | Orchidaceae | Native |
| *Anacamptis pyramidalis* (L.) Rich. | Orchidaceae | Native |
| *Anchusa azurea* Mill. | Boraginaceae | Native |
| *Anchusa officinalis* L. | Boraginaceae | Native |
| *Anthemis arvensis* L. | Asteraceae | Native |
| *Anthemis cretica* subsp. *columnae* (Ten.) Franzén | Asteraceae | Native |
| *Anthoxanthum odoratum* L. | Poaceae | Native |
| *Anthyllis vulneraria* L. | Fabaceae | Native |
| *Aquilegia vulgaris* L. | Ranunculaceae | Native |
| *Arabis collina* Ten. | Brassicaceae | Native |
| *Arabis hirsuta* (L.) Scop. | Brassicaceae | Native |
| *Arctium lappa* L. | Asteraceae | Native |
| *Arctium minus* (Hill) Bernh. | Asteraceae | Native |
| *Aremonia agrimonoides* (L.) DC. | Rosaceae | Native |
| *Arenaria serpyllifolia* L. | Caryophyllaceae | Native |
| *Armeria arenaria* Schult. | Plumbaginaceae | Native |
| *Armeria canescens* (Host) Boiss. | Plumbaginaceae | Native |
| *Armeria canescens* subsp. *nebrodensis* (Guss.) P.Silva | Plumbaginaceae | Native |
| *Arrhenatherum elatius* (L.) P.Beauv. ex J.Presl & C.Presl | Poaceae | Native |
| *Artemisia alba* Turra | Asteraceae | Native |
| *Artemisia verlotiorum* Lamotte | Asteraceae | Neophyte |
| *Artemisia vulgari*s L. | Asteraceae | Native |
| *Asparagus acutifolius* L. | Asparagaceae | Native |
| *Asphodelus macrocarpus* Parl. | Asphodelaceae | Native |
| *Asplenium ceterach* L. | Aspleniaceae | Native |
| *Asplenium trichomanes* L. | Aspleniaceae | Native |
| *Astragalus glycyphyllos* L. | Fabaceae | Native |
| *Astragalus monspessulanus* L. | Fabaceae | Native |
| *Astragalus sempervirens* Lam. | Fabaceae | Native |
| *Atriplex prostrata* Boucher ex DC. | Amaranthaceae | Native |
| *Atropa belladonna* L. | Solanaceae | Native |
| *Avena barbata* Pott ex Link | Poaceae | Native |
| *Avena fatua* L. | Poaceae | Native |
| *Avena sterilis* L. | Poaceae | Native |
| *Ballota nigra* L. | Lamiaceae | Native |
| *Barbarea verna* (Mill.) Asch. | Brassicaceae | Native |
| *Bellardiochloa variegata* (Lam.) Kerguélen | Poaceae | Native |
| *Bellis perennis* L. | Asteraceae | Native |
| *Bellis sylvestris* Cirillo | Asteraceae | Native |
| *Biscutella laevigata* L. | Brassicaceae | Native |
| *Bituminaria bituminosa* (L.) C.H.Stirt. | Fabaceae | Native |
| *Blackstonia perfoliata* (L.) Huds. | Gentianaceae | Native |
| *Bombycilaena erecta* (L.) Smoljan. | Asteraceae | Native |
| *Bothriochloa ischaemum* (L.) Keng | Poaceae | Native |
| *Brachypodium genuense* (DC.) Roem. & Schult. | Poaceae | Native |
| *Brachypodium sylvaticum* (Huds.) P.Beauv. | Poaceae | Native |
| *Briza maxima* L. | Poaceae | Native |
| *Briza media* L. | Poaceae | Native |
| *Briza mino*r L. | Poaceae | Native |
| *Bromus diandrus* Roth | Poaceae | Native |
| *Bromus erectus* Huds. | Poaceae | Native |
| *Bromus hordeaceus* L. | Poaceae | Native |
| *Bromus inermis* Leyss. | Poaceae | Neophyte |
| *Bromus madritensis* L. | Poaceae | Native |
| *Bromus rigidus* Roth | Poaceae | Native |
| *Bromus squarrosus* L. | Poaceae | Native |
| *Bromus sterilis* L. | Poaceae | Native |
| *Bromus tectorum* L. | Poaceae | Native |
| *Buglossoides arvensis* (L.) I.M.Johnst. | Boraginaceae | Native |
| *Bunias erucago* L. | Brassicaceae | Native |
| *Bunium bulbocastanum* L. | Apiaceae | Native |
| *Bupleurum baldense* Turra | Apiaceae | Native |
| *Bupleurum falcatum* subsp. *cernuum* (Nyman) Arcang. | Apiaceae | Native |
| *Bupleurum praealtum* L. | Apiaceae | Native |
| *Bupleurum rotundifolium* L. | Apiaceae | Native |
| *Buxus sempervirens* L. | Buxaceae | Native |
| *Calamagrostis epigejos* (L.) Roth | Poaceae | Native |
| *Calamagrostis varia* (Schrad.) Host | Poaceae | Native |
| *Calystegia sepium* (L.) R.Br. | Convolvulaceae | Native |
| *Campanula erinus* L. | Campanulaceae | Native |
| *Campanula glomerata* L. | Campanulaceae | Native |
| *Campanula rapunculus* L. | Campanulaceae | Native |
| *Campanula scheuchzeri* Vill. | Campanulaceae | Native |
| *Campanula trachelium* L. | Campanulaceae | Native |
| *Capsella bursa-pastoris* Medik. | Brassicaceae | Native |
| *Cardamine bulbifera* Crantz | Brassicaceae | Native |
| *Cardamine heptaphylla* (Vill.) O.E.Schulz | Brassicaceae | Native |
| *Cardamine kitaibelii* Bech. | Brassicaceae | Native |
| *Carduus nutans* L. | Asteraceae | Native |
| *Carduus pycnocephalus* L. | Asteraceae | Native |
| *Carduus tenuiflorus* Curtis | Asteraceae | Native |
| *Carex flacca* Schreb. | Cyperaceae | Native |
| *Carex halleriana* Asso | Cyperaceae | Native |
| *Carex hirta* L. | Cyperaceae | Native |
| *Carex kitaibeliana* Degen ex Bech. | Cyperaceae | Native |
| *Carex macrolepis* DC. | Cyperaceae | Native |
| *Carex spicata* subsp. *spicata* Huds. | Cyperaceae | Native |
| *Carlina acaulis* L. | Asteraceae | Native |
| *Carlina vulgaris* L. | Asteraceae | Native |
| *Carpinus betulus* L. | Betulaceae | Native |
| *Carthamus lanatus* L. | Asteraceae | Native |
| *Catapodium rigidum* (L.) C.E.Hubb. | Poaceae | Native |
| *Cedrus deodara* (Roxb. Ex. D.Don) G.Don | Pinaceae | Neophyte |
| *Celtis australis* L. | Cannabaceae | Native |
| *Centaurea ambigua* Guss. | Asteraceae | Native |
| *Centaurea calcitrapa* L. | Asteraceae | Native |
| *Centaurea delucae* C.Guarino & Rampone | Asteraceae | Native |
| *Centaurea deusta* Ten. | Asteraceae | Native |
| *Centaurea jacea* subsp. *angustifolia* (DC.) | Asteraceae | Native |
| *Centaurea jacea* subsp. *Gaudinii* (Boiss. & Reut.) Gremli | Asteraceae | Native |
| *Centaurea tenoreana* Willk. | Asteraceae | Native |
| *Centaurea triumfettii* All. | Asteraceae | Native |
| *Centaurium erythraea* Rafn | Gentianaceae | Native |
| *Cephalanthera damasonium* Druce | Orchidaceae | Native |
| *Cephalanthera rubra* (L.) Rich. | Orchidaceae | Native |
| *Cerastium arvense* L. | Caryophyllaceae | Native |
| *Cerastium arvense* subsp. *suffruticosum* (L.) Hegi | Caryophyllaceae | Native |
| *Cerastium fontanum* subsp. *Vulgare* (Hartm.) Greuter & Burdet | Caryophyllaceae | Native |
| *Cerastium glomeratum* Thuill. | Caryophyllaceae | Native |
| *Cerastium tomentosum* L. | Caryophyllaceae | Native |
| *Chaerophyllum temulum* L. | Apiaceae | Native |
| *Chamaecytisus hirsutus* (L.) Link | Fabaceae | Native |
| *Chamaecytisus spinescens* Rothm. | Fabaceae | Native |
| *Chenopodium album* L. | Amaranthaceae | Native |
| *Chenopodium vulvaria* L. | Amaranthaceae | Native |
| *Chondrilla juncea* L. | Asteraceae | Native |
| *Cichorium intybus* L. | Asteraceae | Native |
| *Cirsium arvense* (L.) Scop. | Asteraceae | Native |
| *Cirsium eriophorum* Scop. | Asteraceae | Native |
| *Cirsium vulgare* (Savi) Ten. | Asteraceae | Native |
| *Cistus creticus* L. | Cistaceae | Native |
| *Clematis flammula* L. | Ranunculaceae | Native |
| *Clematis vitalba* L. | Ranunculaceae | Native |
| *Clinopodium acinos* Kuntze | Lamiaceae | Native |
| *Clinopodium alpinum* Kuntze | Lamiaceae | Native |
| *Clinopodium nepeta* (L.) Kuntze | Lamiaceae | Native |
| *Clinopodium vulgare* L. | Lamiaceae | Native |
| *Convolvulus althaeoides* subsp. *tenuissimus* (Sm.) Batt. (Sm.) Batt. | Convolvulaceae | Native |
| *Convolvulus arvensis* L. | Convolvulaceae | Native |
| *Convolvulus cantabrica* L. | Convolvulaceae | Native |
| *Coreopsis lanceolata* L. | Asteraceae | Neophyte |
| *Cornus mas* L. | Cornaceae | Native |
| *Cornus sanguinea* L. | Cornaceae | Native |
| *Coronilla minima* L. | Fabaceae | Native |
| *Coronilla scorpioides* (L.) Koch | Fabaceae | Native |
| *Coronilla vaginalis* Lam. | Fabaceae | Native |
| *Coronilla varia* L. | Fabaceae | Native |
| *Corylus avellana* L. | Betulaceae | Native |
| *Cota tinctoria* (L.) J.Gay | Asteraceae | Native |
| *Cotoneaster tomentosus* (Aiton) Lindl. | Rosaceae | Native |
| *Crataegus laevigata* (Poir.) DC. | Rosaceae | Native |
| *Crataegus monogyna* Jacq. | Rosaceae | Native |
| *Crataegus rhipidophylla* Gand. | Rosaceae | Native |
| *Crepis biennis* L. | Asteraceae | Native |
| *Crepis capillaris* (L.) Wallr. | Asteraceae | Native |
| *Crepis foetida* subsp. *foetida* L. | Asteraceae | Native |
| *Crepis lacera* Ten. | Asteraceae | Native |
| *Crepis neglecta* L. | Asteraceae | Native |
| *Crepis pulchra* subsp. *pulchra* L. | Asteraceae | Native |
| *Crepis sancta* (L.) Babc. | Asteraceae | Native |
| *Crepis setosa* Haller f. | Asteraceae | Native |
| *Crepis tectorum* L. | Asteraceae | Native |
| *Crepis vesicaria* L. | Asteraceae | Native |
| *Cruciata glabra* (L.) Opiz | Rubiaceae | Native |
| *Cruciata laevipes* Opiz | Rubiaceae | Native |
| *Cyclamen hederifolium* Aiton | Primulaceae | Native |
| *Cymbalaria muralis* G.Gaertn., B.Mey. & Scherb. | Plantaginaceae | Native |
| *Cynanchica aristata* (L.f.) P.Caputo & Del Guacchio | Rubiaceae | Native |
| *Cynanchica pyrenaica* subsp. *cynanchica* (L.) P.Caputo & Del Guacchio | Rubiaceae | Native |
| *Cynara cardunculus* L. | Asteraceae | Native |
| *Cynodon dactylon* (L.) Pers. | Poaceae | Native |
| *Cynoglossum magellense* Ten. | Boraginaceae | Native |
| *Cynoglossum officinale* L. | Boraginaceae | Native |
| *Cynosurus cristatus* L. | Poaceae | Native |
| *Cynosurus echinatus* L. | Poaceae | Native |
| *Cytisophyllum sessilifolium* (L.) O.Lang | Fabaceae | Native |
| *Cytisus villosus* Pourr. | Fabaceae | Native |
| *Dactylis glomerata* L. | Poaceae | Native |
| *Dactylorhiza maculata* (L.) Soó | Orchidaceae | Native |
| *Daphne laureola* L. | Thymelaeaceae | Native |
| *Dasypyrum villosum* (L.) Borbás | Poaceae | Native |
| *Daucus carota* L. | Apiaceae | Native |
| *Delphinium consolida* L. | Ranunculaceae | Native |
| *Dianthus brachycalyx* A.Huet & É.Huet ex Bacch., Brullo, Casti & Giusso | Caryophyllaceae | Native |
| *Dianthus carthusianorum* L. | Caryophyllaceae | Native |
| *Dianthus monspessulanus* L. | Caryophyllaceae | Native |
| *Dianthus sylvestris* Wulfen | Caryophyllaceae | Native |
| *Digitalis lutea* L. | Plantaginaceae | Native |
| *Digitalis lutea* subsp. *australis* (Ten.) Arcang. | Plantaginaceae | Native |
| *Dipsacus fullonum* L. | Caprifoliaceae | Native |
| *Dittrichia viscosa* subsp. *viscosa* (L.) Greuter | Asteraceae | Native |
| *Draba verna* L. | Brassicaceae | Native |
| *Echinops sphaerocephalus* L. | Asteraceae | Native |
| *Echium plantagineum* L. | Boraginaceae | Native |
| *Echium vulgare* L. | Boraginaceae | Native |
| *Elymus caninus* (L.) L. | Poaceae | Native |
| *Elymus repens* (L.) Gould | Poaceae | Native |
| *Epilobium montanum* L. | Onagraceae | Native |
| *Epilobium tetragonum* L. | Onagraceae | Native |
| *Epipactis helleborine* (L.) Crantz | Orchidaceae | Native |
| *Erigeron annuus* (L.) Desf. | Asteraceae | Neophyte |
| *Erigeron canadensis* L. | Asteraceae | Neophyte |
| *Erigeron sumatrensis* Retz. | Asteraceae | Neophyte |
| *Erodium botrys* (Cav.) Bertol. | Geraniaceae | Native |
| *Erodium cicutarium* (L.) L’Hér. | Geraniaceae | Native |
| *Erodium malacoides* (L.) L’Hér. | Geraniaceae | Native |
| *Eryngium amethystinum* L. | Apiaceae | Native |
| *Eryngium campestre* L. | Apiaceae | Native |
| *Erysimum pseudorhaeticum* Polatschek | Brassicaceae | Native |
| *Euonymus europaeus* L. | Celastraceae | Native |
| *Euonymus latifolius* (L.) Mill. | Celastraceae | Native |
| *Eupatorium cannabinum* L. | Asteraceae | Native |
| *Euphorbia amygdaloides* L. | Euphorbiaceae | Native |
| *Euphorbia cyparissias* L. | Euphorbiaceae | Native |
| *Euphorbia exigua* L. | Euphorbiaceae | Native |
| *Euphorbia helioscopia* L. | Euphorbiaceae | Native |
| *Euphorbia hirsuta* L. | Euphorbiaceae | Native |
| *Euphorbia myrsinites* L. | Euphorbiaceae | Native |
| *Euphorbia prostrata* Aiton | Euphorbiaceae | Neophyte |
| *Euphrasia officinalis* subsp. *pratensis* Fr. | Orobanchaceae | Native |
| *Euphrasia stricta* J.P.Wolff ex J.F.Lehm. | Orobanchaceae | Native |
| *Fagus sylvatica* L. | Fagaceae | Native |
| *Festuca circummediterranea* Patzke | Poaceae | Native |
| *Festuca dimorpha* Guss. | Poaceae | Native |
| *Festuca heterophylla* Lam. | Poaceae | Native |
| *Festuca inops* De Not. | Poaceae | Native |
| *Festuca myuros* L. | Poaceae | Native |
| *Foeniculum vulgare* Mill. | Apiaceae | Native |
| *Fragaria vesca* L. | Rosaceae | Native |
| *Fraxinus excelsior* L. | Oleaceae | Native |
| *Fraxinus ornus* L. | Oleaceae | Native |
| *Fumana procumbens* Gren. & Godr. | Cistaceae | Native |
| *Fumaria capreolata* L. | Papaveraceae | Native |
| *Fumaria vaillantii* Loisel. | Papaveraceae | Native |
| *Galactites tomentosus* Moench | Asteraceae | Native |
| *Galeopsis angustifolia* Ehrh. Ex Hoffm. | Lamiaceae | Native |
| *Galeopsis tetrahit* L. | Lamiaceae | Native |
| *Galium aparine* L. | Rubiaceae | Native |
| *Galium lucidum* All. | Rubiaceae | Native |
| *Galium mollugo* L. | Rubiaceae | Native |
| *Galium odoratum* Scop. | Rubiaceae | Native |
| *Galium rotundifolium* L. | Rubiaceae | Native |
| *Galium sylvaticum* L. | Rubiaceae | Native |
| *Galium verum* L. | Rubiaceae | Native |
| *Genista tinctoria* L. | Fabaceae | Native |
| *Gentiana acaulis* subsp. *dinarica* (Beck) Barina | Gentianaceae | Native |
| *Gentiana verna* L. | Gentianaceae | Native |
| *Geranium columbinum* L. | Geraniaceae | Native |
| *Geranium dissectum* L. | Geraniaceae | Native |
| *Geranium lucidum* L. | Geraniaceae | Native |
| *Geranium purpureum* Vill. | Geraniaceae | Native |
| *Geranium pusillum* L. | Geraniaceae | Native |
| *Geranium pyrenaicum* Burm.f. | Geraniaceae | Native |
| *Geranium rotundifolium* L. | Geraniaceae | Native |
| *Geranium sanguineum* L. | Geraniaceae | Native |
| *Geum rivale* L. | Rosaceae | Native |
| *Geum urbanum* L. | Rosaceae | Native |
| *Gladiolus italicus* Mill. | Iridaceae | Native |
| *Globularia bisnagarica* L. | Plantaginaceae | Native |
| *Globularia meridionalis* (Podp.) O.Schwarz | Plantaginaceae | Native |
| *Hedera helix* L. | Araliaceae | Native |
| *Helianthemum apenninum* Mill. | Cistaceae | Native |
| *Helianthemum canum* (L.) Hornem. | Cistaceae | Native |
| *Helianthemum nummularium* subsp. *grandiflorum* (Scop.) Schinz & Thell. | Cistaceae | Native |
| *Helianthemum nummularium* subsp. *obscurum* (Pers.) Holub | Cistaceae | Native |
| *Helichrysum italicum* (Roth) G.Don | Asteraceae | Native |
| *Helictochloa versicolor* subsp. *praetutiana* (Parl. Ex Arcang.) Romero Zarco | Poaceae | Native |
| *Helleborus foetidus* L. | Ranunculaceae | Native |
| *Hepatica nobilis* Schreb. | Ranunculaceae | Native |
| *Herniaria hirsuta* L. | Caryophyllaceae | Native |
| *Hesperocyparis arizonica* (Greene) Bartel | Cupressaceae | Neophyte |
| *Hieracium murorum* L. | Asteraceae | Native |
| *Hieracium pilosum* Froel. | Asteraceae | Native |
| *Hippocrepis biflora* Spreng. | Fabaceae | Native |
| *Hippocrepis comosa* L. | Fabaceae | Native |
| *Hippocrepis emerus* subsp. *emerus* (L.) Lassen | Fabaceae | Native |
| *Holcus lanatus* L. | Poaceae | Native |
| *Hordelymus europaeus* (L.) Jess. Ex Harz | Poaceae | Native |
| *Hordeum bulbosum* L. | Poaceae | Native |
| *Hordeum murinum* L. | Poaceae | Native |
| *Hordeum vulgare* L. | Poaceae | Native |
| *Humulus lupulus* L. | Cannabaceae | Native |
| *Hypericum perfoliatum* L. | Hypericaceae | Native |
| *Hypericum perforatum* L. | Hypericaceae | Native |
| *Hypericum tetrapterum* Fr. | Hypericaceae | Native |
| *Hypochaeris achyrophorus* L. | Asteraceae | Native |
| *Hypochaeris cretensis* (L.) Bory & Chaub. | Asteraceae | Native |
| *Hypochaeris radicata* L. | Asteraceae | Native |
| *Hyssopus officinalis* L. | Lamiaceae | Native |
| *Inula conyza* DC. | Asteraceae | Native |
| *Inula montana* L. | Asteraceae | Native |
| *Iris* x *germanica* L. | Iridaceae | Native |
| *Isatis tinctoria* L. | Brassicaceae | Archaeophyte |
| *Jacobaea erucifolia* (L.) G.Gaertn., B.Mey. & Scherb. | Asteraceae | Native |
| *Jasione montana* subsp. *montana* L. | Campanulaceae | Native |
| *Juglans regia* L. | Juglandaceae | Native |
| *Juniperus communis* L. | Cupressaceae | Native |
| *Juniperus deltoides* R.P.Adams | Cupressaceae | Native |
| *Knautia calycina* (C.Presl) Guss. | Caprifoliaceae | Native |
| *Knautia collina* (Gaudin) Jord. | Caprifoliaceae | Native |
| *Koeleria splendens* C.Presl | Poaceae | Native |
| *Laburnum anagyroidis* Medik. | Fabaceae | Native |
| *Lactuca muralis* (L.) Gaertn. | Asteraceae | Native |
| *Lactuca perennis* L. | Asteraceae | Native |
| *Lactuca serriola* L. | Asteraceae | Native |
| *Lactuca viminea* (L.) J.Presl & C.Presl | Asteraceae | Native |
| *Lamium maculatum* L. | Lamiaceae | Native |
| *Lamium purpureum* L. | Lamiaceae | Native |
| *Lapsana communis* L. | Asteraceae | Native |
| *Lathyrus aphaca* L. | Fabaceae | Native |
| *Lathyrus cicera* L. | Fabaceae | Native |
| *Lathyrus hirsutus* L. | Fabaceae | Native |
| *Lathyrus inconspicuus* L. | Fabaceae | Native |
| *Lathyrus latifolius* L. | Fabaceae | Native |
| *Lathyrus pratensis* L. | Fabaceae | Native |
| *Lathyrus sphaericus* Retz. | Fabaceae | Native |
| *Lathyrus sylvestris* L. | Fabaceae | Native |
| *Lathyrus venetus* (Mill.) Wohlf. | Fabaceae | Native |
| *Lathyrus vernus* (L.) Bernh. | Fabaceae | Native |
| *Legousia speculum-veneris* (L.) Chaix | Campanulaceae | Native |
| *Leontodon crispus* Vill. | Asteraceae | Native |
| *Leontodon hispidus* L. | Asteraceae | Native |
| *Leontodon rosani* (Ten.) DC. | Asteraceae | Native |
| *Leopoldia comosa* (L.) Parl. | Asparagaceae | Native |
| *Leucanthemum pallens* DC. | Asteraceae | Native |
| *Ligustrum vulgare* L. | Oleaceae | Native |
| *Limodorum abortivum* (L.) Sw. | Orchidaceae | Native |
| *Linaria purpurea* (L.) Mill. | Plantaginaceae | Native |
| *Linaria vulgaris* Mill. | Plantaginaceae | Native |
| *Linum bienne* Mill. | Linaceae | Native |
| *Linum catharticum* L. | Linaceae | Native |
| *Linum strictum* L. | Linaceae | Native |
| *Linum tenuifolium* L. | Linaceae | Native |
| *Linum trigynum* L. | Linaceae | Native |
| *Linum usitatissimum* L. | Linaceae | Native |
| *Lithospermum officinale* L. | Boraginaceae | Native |
| *Lolium arundinaceum* (Schreb.) Darbysh. | Poaceae | Native |
| *Lolium perenne* L. | Poaceae | Native |
| *Lolium pratense* (Huds.) Darbysh. | Poaceae | Native |
| *Lonicera caprifolium* L. | Caprifoliaceae | Native |
| *Lonicera etrusca* Santi | Caprifoliaceae | Native |
| *Lonicera implexa* Aiton | Caprifoliaceae | Native |
| *Lonicera xylosteum* L. | Caprifoliaceae | Native |
| *Lotus corniculatus* L. | Fabaceae | Native |
| *Lotus dorycnium* L. | Fabaceae | Native |
| *Lotus edulis* L. | Fabaceae | Native |
| *Lotus hirsutus* L. | Fabaceae | Native |
| *Lotus parviflorus* Desf. | Fabaceae | Native |
| *Lunaria annua* L. | Brassicaceae | Native |
| *Luzula campestris* (L.) DC. | Juncaceae | Native |
| *Luzula sylvatica* subsp. *sieberi* (Tausch) K.Richt. | Juncaceae | Native |
| *Lysimachia arvensis* (L.) U.Manns & Anderb. | Primulaceae | Native |
| *Malus domestica* (Suckow) Borkh. | Rosaceae | Native |
| *Malus sylvestris* (L.) Mill. | Rosaceae | Native |
| *Malva setigera* K.F.Schimp. & Spenn. | Malvaceae | Native |
| *Malva sylvestris* L. | Malvaceae | Native |
| *Matthiola fruticulosa* (L.) Maire | Brassicaceae | Native |
| *Medicago falcata* L. | Fabaceae | Native |
| *Medicago lupulina* L. | Fabaceae | Native |
| *Medicago minima* (L.) Bartal. | Fabaceae | Native |
| *Medicago orbicularis* (L.) Bartal. | Fabaceae | Native |
| *Medicago polymorpha* L. | Fabaceae | Native |
| *Medicago rigidula* (L.) All. | Fabaceae | Native |
| *Medicago sativa* L. | Fabaceae | Native |
| *Melampyrum italicum* (Beauverd) Soó | Orobanchaceae | Native |
| *Melica ciliata* L. | Poaceae | Native |
| *Melica uniflora* Retz. | Poaceae | Native |
| *Melilotus albus* Medik. | Fabaceae | Native |
| *Melilotus altissimus* Thuill. | Fabaceae | Native |
| *Melilotus officinalis* (L.) Pall. | Fabaceae | Native |
| *Melissa officinalis* L. | Lamiaceae | Native |
| *Mentha longifolia* (L.) Huds. | Lamiaceae | Native |
| *Mentha suaveolens* Ehrh. | Lamiaceae | Native |
| *Mercurialis perennis* L. | Euphorbiaceae | Native |
| *Micromeria graeca* (L.) Benth. Ex Rchb. | Lamiaceae | Native |
| *Minuartia graminifolia* Javorka | Caryophyllaceae | Native |
| *Muscari neglectum* Guss. Ex Ten. | Asparagaceae | Native |
| *Myosotis arvensis* Hill | Boraginaceae | Native |
| *Myosotis stricta* Link ex Roem. & Schult. | Boraginaceae | Native |
| *Nigella damascena* L. | Ranunculaceae | Native |
| *Odontites luteus* (L.) Clairv. | Orobanchaceae | Native |
| *Olea europaea* L. | Oleaceae | Native |
| *Oloptum miliaceum* (L.) Röser & Hamasha | Poaceae | Native |
| *Onobrychis viciifolia* Scop. | Fabaceae | Native |
| *Ononis pusilla* L. | Fabaceae | Native |
| *Ophrys bertolonii* Moretti | Orchidaceae | Native |
| *Ophrys fusca* Link | Orchidaceae | Native |
| *Ophrys holosericea* subsp. *holosericea* (Burm.f.) Greuter | Orchidaceae | Native |
| *Orchis purpurea* Huds. | Orchidaceae | Native |
| *Origanum vulgare* L. | Lamiaceae | Native |
| *Orlaya grandiflora* (L.) Hoffm. | Apiaceae | Native |
| *Ornithogalum divergens* Boreau | Asparagaceae | Native |
| *Ornithogalum pyramidale* L. | Asparagaceae | Native |
| *Ornithogalum umbellatum* L. | Asparagaceae | Native |
| *Ostrya carpinifolia* Scop. | Betulaceae | Native |
| *Osyris alba* L. | Santalaceae | Native |
| *Pallenis spinosa* subsp. *spinosa* (L.) Cass. | Asteraceae | Native |
| *Papaver dubium* L. | Papaveraceae | Native |
| *Papaver rhoeas* L. | Papaveraceae | Native |
| *Parentucellia latifolia* (L.) Caruel | Orobanchaceae | Native |
| *Parietaria judaica* L. | Urticaceae | Native |
| *Patzkea paniculata* (L.) G.H.Loos | Poaceae | Native |
| *Pedicularis elegans* Ten. | Orobanchaceae | Native |
| *Petasites albus* (L.) Gaertn. | Asteraceae | Native |
| *Petrorhagia prolifera* (L.) P.W.Ball & Heywood | Caryophyllaceae | Native |
| *Petrorhagia saxifraga* Link | Caryophyllaceae | Native |
| *Petrosedum rupestre* (L.) P.V.Heath | Crassulaceae | Native |
| *Petrosedum sediforme* (Jacq.) Grulich | Crassulaceae | Native |
| *Phleum hirsutum* Honck. | Poaceae | Native |
| *Phleum pratense* L. | Poaceae | Native |
| *Picris hieracioides* L. | Asteraceae | Native |
| *Pilosella officinarum* Vaill. | Asteraceae | Native |
| *Pilosella piloselloides* (Vill.) Soják | Asteraceae | Native |
| *Pimpinella saxifraga* L. | Apiaceae | Native |
| *Pimpinella tragium* Vill. | Apiaceae | Native |
| *Pinus nigra* J.F.Arnold | Pinaceae | Native |
| *Pistacia terebinthus* L. | Anacardiaceae | Native |
| *Plantago argentea* subsp. *argentea* Chaix | Plantaginaceae | Native |
| *Plantago lagopus* L. | Plantaginaceae | Native |
| *Plantago lanceolata* L. | Plantaginaceae | Native |
| *Plantago major* L. | Plantaginaceae | Native |
| *Plantago media* L. | Plantaginaceae | Native |
| *Plantago strictissima* L. | Plantaginaceae | Native |
| *Plantago subulata* L. | Plantaginaceae | Native |
| *Platanthera bifolia* (L.) Rich. | Orchidaceae | Native |
| *Platanthera chlorantha* (Custer) Rchb. | Orchidaceae | Native |
| *Plumbago europaea* L. | Plumbaginaceae | Native |
| *Poa alpina* L. | Poaceae | Native |
| *Poa angustifolia* L. | Poaceae | Native |
| *Poa annua* L. | Poaceae | Native |
| *Poa bulbosa* L. | Poaceae | Native |
| *Poa molinerii* Balb. | Poaceae | Native |
| *Poa nemoralis* L. | Poaceae | Native |
| *Poa pratensis* L. | Poaceae | Native |
| *Poa trivialis* L. | Poaceae | Native |
| *Podospermum canum* C.A.Mey. | Asteraceae | Native |
| *Podospermum laciniatum* subsp. *laciniatum* (L.) DC. | Asteraceae | Native |
| *Polygala flavescens* DC. | Polygalaceae | Native |
| *Polygala major* Jacq. | Polygalaceae | Native |
| *Polygala nicaeensis* Risso ex W.D.J.Koch | Polygalaceae | Native |
| *Polygonum aviculare* L. | Polygonaceae | Native |
| *Polypogon viridis* (Gouan) Breistr. | Poaceae | Native |
| *Polystichum aculeatum* (L.) Roth | Dryopteridaceae | Native |
| *Populus alba* L. | Salicaceae | Native |
| *Populus nigra* L. | Salicaceae | Native |
| *Portulaca oleracea* L. | Portulacaceae | Native |
| *Potentilla erecta* (L.) Raeusch. | Rosaceae | Native |
| *Potentilla heptaphylla* L. | Rosaceae | Native |
| *Potentilla recta* L. | Rosaceae | Native |
| *Potentilla reptans* L. | Rosaceae | Native |
| *Potentilla rigoana* Th.Wolf | Rosaceae | Native |
| *Prenanthes purpurea* L. | Asteraceae | Native |
| *Primula veris* L. | Primulaceae | Native |
| *Primula vulgaris* Huds. | Primulaceae | Native |
| *Prunella grandiflora* (L.) Turra | Lamiaceae | Native |
| *Prunella vulgaris* L. | Lamiaceae | Native |
| *Prunus avium* (L.) L. | Rosaceae | Native |
| *Prunus cerasus* L. | Rosaceae | Native |
| *Prunus domestica* L. | Rosaceae | Native |
| *Prunus dulcis* D.A.Webb | Rosaceae | Native |
| *Prunus mahaleb* L. | Rosaceae | Native |
| *Prunus spinosa* L. | Rosaceae | Native |
| *Pseudoturritis turrita* (L.) Al-Shehbaz | Brassicaceae | Native |
| *Pteridium aquilinum* (L.) Kuhn | Dennstaedtiaceae | Native |
| *Pulicaria odora* (L.) Rchb. | Asteraceae | Native |
| *Pulmonaria vallarsae* subsp. *apennina* (Cristof. & Puppi) L.Cecchi & Selvi | Boraginaceae | Native |
| *Pyracantha coccinea* M.Roem. | Rosaceae | Native |
| *Pyrus pyraster* (L.) Burgsd. | Rosaceae | Native |
| *Quercus cerris* L. | Fagaceae | Native |
| *Quercus ilex* L. | Fagaceae | Native |
| *Quercus pubescens* Willd. | Fagaceae | Native |
| *Ranunculus acris* L. | Ranunculaceae | Native |
| *Ranunculus bulbosus* L. | Ranunculaceae | Native |
| *Ranunculus flammula* L. | Ranunculaceae | Native |
| *Ranunculus lanuginosus* L. | Ranunculaceae | Native |
| *Reichardia picroides* (L.) Roth | Asteraceae | Native |
| *Reseda phyteuma* L. | Resedaceae | Native |
| *Rhagadiolus stellatus* (L.) Gaertn. | Asteraceae | Native |
| *Rhamnus alpina* L. | Rhamnaceae | Native |
| *Rhinanthus alectorolophus* (Scop.) Pollich | Orobanchaceae | Native |
| *Rhinanthus minor* L. | Orobanchaceae | Native |
| *Rhinanthus wettsteinii* (Sterneck) Soó | Orobanchaceae | Native |
| *Robinia pseudoacacia* L. | Fabaceae | Neophyte |
| *Rosa agrestis* Savi | Rosaceae | Native |
| *Rosa canina* L. | Rosaceae | Native |
| *Rosa micrantha* Borrer | Rosaceae | Native |
| *Rosa pendulina* L. | Rosaceae | Native |
| *Rostraria cristata* (L.) Tzvelev | Poaceae | Native |
| *Rubus caesius* L. | Rosaceae | Native |
| *Rubus hirtus* Waldst. & Kit. | Rosaceae | Native |
| *Rubus idaeus* L. | Rosaceae | Native |
| *Rubus incanescens* Bertol. | Rosaceae | Native |
| *Rubus ulmifolius* Schott | Rosaceae | Native |
| *Rumex acetosa* L. | Polygonaceae | Native |
| *Rumex conglomeratus* Murray | Polygonaceae | Native |
| *Rumex crispus* L. | Polygonaceae | Native |
| *Rumex pulcher* L. | Polygonaceae | Native |
| *Rumex scutatus* L. | Polygonaceae | Native |
| *Ruscus aculeatus* L. | Asparagaceae | Native |
| *Salvia glutinosa* L. | Lamiaceae | Native |
| *Salvia pratensis* L. | Lamiaceae | Native |
| *Salvia verbenaca* L. | Lamiaceae | Native |
| *Sambucus ebulus* L. | Viburnaceae | Native |
| *Sanguisorba minor* Scop. | Rosaceae | Native |
| *Sanicula europaea* L. | Apiaceae | Native |
| *Saponaria ocymoides* L. | Caryophyllaceae | Native |
| *Saponaria officinalis* L. | Caryophyllaceae | Native |
| *Satureja montana* L. | Lamiaceae | Native |
| *Scabiosa atropurpurea* L. | Caprifoliaceae | Native |
| *Scabiosa columbaria* L. | Caprifoliaceae | Native |
| *Scabiosa columbaria* subsp. *portae* (A.Kern. ex Huter) Hayek | Caprifoliaceae | Native |
| *Scabiosa holosericea* Bertol. | Caprifoliaceae | Native |
| *Scilla bifolia* L. | Asparagaceae | Native |
| *Scorpiurus muricatus* L. | Fabaceae | Native |
| *Scrophularia canina* L. | Scrophulariaceae | Native |
| *Scrophularia peregrina* L. | Scrophulariaceae | Native |
| *Scutellaria columnae* All. | Lamiaceae | Native |
| *Sedum acre* L. | Crassulaceae | Native |
| *Sedum album* L. | Crassulaceae | Native |
| *Sedum hispanicum* L. | Crassulaceae | Native |
| *Sedum rubens* L. | Crassulaceae | Native |
| *Sedum sexangulare* L. | Crassulaceae | Native |
| *Senecio inaequidens* DC. | Asteraceae | Neophyte |
| *Senecio ovatus* subsp. *stabianus* (Lacaita) Greuter | Asteraceae | Native |
| *Seseli montanum* L. | Apiaceae | Native |
| *Seseli montanum* subsp. *tommasinii* (Rchb.f.) Arcang. | Apiaceae | Native |
| *Sesleria nitida* Ten. | Poaceae | Native |
| *Setaria pumila* (Poir.) Roem. & Schult. | Poaceae | Native |
| *Sherardia arvensis* L. | Rubiaceae | Native |
| *Sideritis montana* L. | Lamiaceae | Native |
| *Sideritis romana* L. | Lamiaceae | Native |
| *Silene ciliata* Pourr. | Caryophyllaceae | Native |
| *Silene dioica* (L.) Clairv. | Caryophyllaceae | Native |
| *Silene italica* (L.) Pers. | Caryophyllaceae | Native |
| *Silene latifolia* Poir. | Caryophyllaceae | Native |
| *Silene multicaulis* Guss. | Caryophyllaceae | Native |
| *Silene noctiflora* L. | Caryophyllaceae | Native |
| *Silene nutans* L. | Caryophyllaceae | Native |
| *Silene otites* (L.) Wibel | Caryophyllaceae | Native |
| *Silene vulgaris* (Moench) Garcke | Caryophyllaceae | Native |
| *Sisymbrium officinale* (L.) Scop. | Brassicaceae | Native |
| *Smilax aspera* L. | Smilacaceae | Native |
| *Sonchus arvensis* subsp. *arvensis* L. | Asteraceae | Native |
| *Sonchus asper* (L.) Hill | Asteraceae | Native |
| *Sonchus oleraceus* L. | Asteraceae | Native |
| *Sorbus aria* (L.) Crantz | Rosaceae | Native |
| *Sorbus domestica* L. | Rosaceae | Native |
| *Sorbus torminalis* (L.) Crantz | Rosaceae | Native |
| *Spartium junceum* L. | Fabaceae | Native |
| *Stachys alopecuros* subsp. *divulsa* (Ten.) Grande | Lamiaceae | Native |
| *Stachys cretica* subsp. *salviifolia* (Ten.) Rech.f. | Lamiaceae | Native |
| *Stachys germanica* L. | Lamiaceae | Native |
| *Stachys officinalis* (L.) Trevis. | Lamiaceae | Native |
| *Stachys recta* L. | Lamiaceae | Native |
| *Stellaria media* (L.) Vill. | Caryophyllaceae | Native |
| *Stellaria nemorum* L. | Caryophyllaceae | Native |
| *Stipa apertifolia* Martinovský | Poaceae | Native |
| *Tanacetum corymbosum* subsp. *corymbosum* (L.) Sch.Bip. | Asteraceae | Native |
| *Taraxacum apenninum* DC. | Asteraceae | Native |
| *Taraxacum officinale* F.H.Wigg. | Asteraceae | Native |
| *Teucrium botrys* L. | Lamiaceae | Native |
| *Teucrium capitatum* L. | Lamiaceae | Native |
| *Teucrium chamaedrys* L. | Lamiaceae | Native |
| *Teucrium montanum* L. | Lamiaceae | Native |
| *Teucrium siculum* Guss. | Lamiaceae | Native |
| *Thalictrum aquilegiifolium* L. | Ranunculaceae | Native |
| *Thalictrum flavum* L. | Ranunculaceae | Native |
| *Thapsia asclepium* L. | Apiaceae | Native |
| *Thesium humifusum* DC. | Santalaceae | Native |
| *Thliphthisa purpurea* (L.) P.Caputo & Del Guacchio | Rubiaceae | Native |
| *Thymus longicaulis* subsp. *longicaulis* C.Presl | Lamiaceae | Native |
| *Thymus pulegioides* L. | Lamiaceae | Native |
| *Thymus striatus* Vahl | Lamiaceae | Native |
| *Tolpis staticifolia* (All.) Sch.Bip. | Asteraceae | Native |
| *Tordylium apulum* L. | Apiaceae | Native |
| *Tordylium maximum* L. | Apiaceae | Native |
| *Torilis africana* Spreng. | Apiaceae | Native |
| *Torilis japonica* DC. | Apiaceae | Native |
| *Tragopogon dubius* Scop. | Asteraceae | Native |
| *Tragopogon porrifolius* L. | Asteraceae | Native |
| *Tragopogon pratensis* L. | Asteraceae | Native |
| *Trifolium alpestre* L. | Fabaceae | Native |
| *Trifolium angustifolium* L. | Fabaceae | Native |
| *Trifolium arvense* L. | Fabaceae | Native |
| *Trifolium campestre* Schreb. | Fabaceae | Native |
| *Trifolium incarnatum* L. | Fabaceae | Native |
| *Trifolium medium* L. | Fabaceae | Native |
| *Trifolium montanum* subsp. *montanum* L. | Fabaceae | Native |
| *Trifolium nigrescens* subsp. *Nigrescens* Viv. | Fabaceae | Native |
| *Trifolium ochroleucon* Huds. | Fabaceae | Native |
| *Trifolium pallescens* Schreb. | Fabaceae | Native |
| *Trifolium pratense* subsp. *pratense* L. | Fabaceae | Native |
| *Trifolium repens* L. | Fabaceae | Native |
| *Trifolium scabrum* L. | Fabaceae | Native |
| *Trifolium stellatum* L. | Fabaceae | Native |
| *Trinia glauca* Rchb. | Apiaceae | Native |
| *Trisetaria aurea* (Ten.) Pignatti | Poaceae | Native |
| *Trisetum flavescens* (L.) P.Beauv. | Poaceae | Native |
| *Triticum aestivum* L. | Poaceae | Native |
| *Tussilago farfara* L. | Asteraceae | Native |
| *Tyrimnus leucographus* Cass. | Asteraceae | Native |
| *Ulmus glabra* Huds. | Ulmaceae | Native |
| *Ulmus minor* Mill. | Ulmaceae | Native |
| *Umbilicus horizontalis* DC. | Crassulaceae | Native |
| *Urospermum dalechampii* (L.) Scop. Ex F.W.Schmidt | Asteraceae | Native |
| *Urtica dioica* L. | Urticaceae | Native |
| *Urtica membranacea* Poir. Ex Savigny | Urticaceae | Native |
| *Valeriana dioica* L. | Caprifoliaceae | Native |
| *Valeriana locusta* L. | Caprifoliaceae | Native |
| *Valeriana officinalis* L. | Caprifoliaceae | Native |
| *Valeriana rubra* L. | Caprifoliaceae | Native |
| *Verbascum phlomoides* L. | Scrophulariaceae | Native |
| *Verbascum pulverulentum* Vill. | Scrophulariaceae | Native |
| *Verbascum sinuatum* L. | Scrophulariaceae | Native |
| *Verbena officinalis* L. | Verbenaceae | Native |
| *Veronica arvensis* L. | Plantaginaceae | Native |
| *Veronica cymbalaria* Bodard | Plantaginaceae | Native |
| *Veronica officinalis* L. | Plantaginaceae | Native |
| *Veronica verna* L. | Plantaginaceae | Native |
| *Viburnum lantana* L. | Viburnaceae | Native |
| *Vicia cracca* L. | Fabaceae | Native |
| *Vicia hirsuta* (L.) Gray | Fabaceae | Native |
| *Vicia hybrida* L. | Fabaceae | Native |
| *Vicia lenticula* (Hoppe) Janka | Fabaceae | Native |
| *Vicia sativa* L. | Fabaceae | Native |
| *Vicia sepium* L. | Fabaceae | Native |
| *Vicia villosa* Roth | Fabaceae | Native |
| *Vicia villosa* subsp. *varia* (Host) Corb. | Fabaceae | Native |
| *Vincetoxicum hirundinaria* Medik. | Apocynaceae | Native |
| *Viola alba* Besser | Violaceae | Native |
| *Viola alba* subsp. *dehnhardtii* (Ten.) W.Becker | Violaceae | Native |
| *Viola arvensis* Murray | Violaceae | Native |
| *Viola hirta* L. | Violaceae | Native |
| *Viola odorata* L. | Violaceae | Native |
| *Viola reichenbachiana* Jord. Ex Boreau | Violaceae | Native |


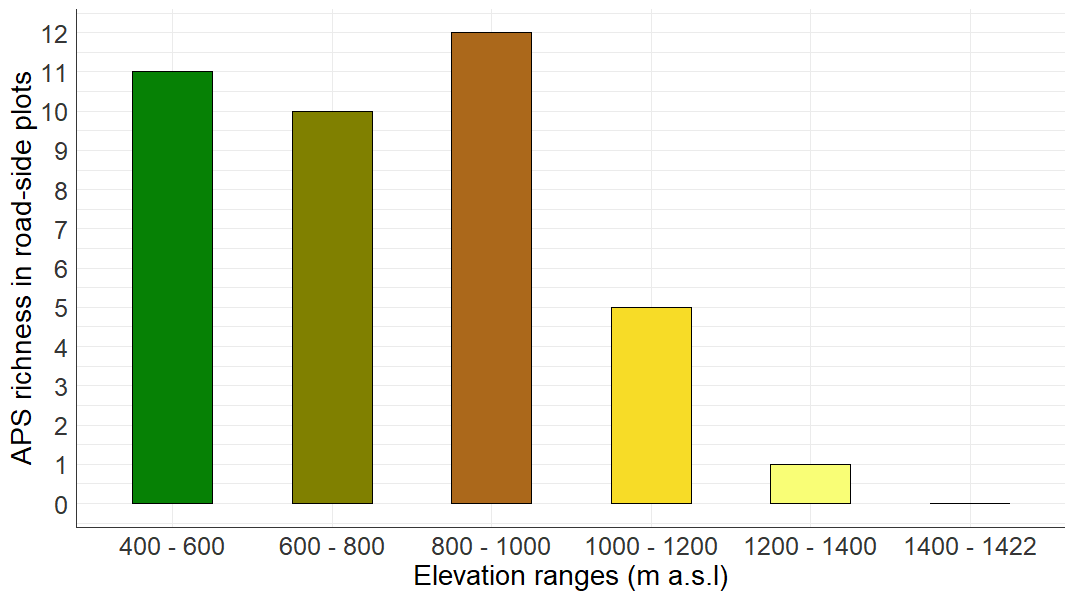


***Fig. S1****. The bar chart shows the Alien Plant Species (APS) richness in road-side plots along the elevation gradient ranges of the study area.*

**Table S4** The table shows the plant species representing about 45% of the total coverage of the road-side plots. The species are ordered with their percentage (%) of total cover in the road-side plots, their families, their status (native/alien) (Galasso et al. 2018; Bartolucci et al. 2021, 2022) and the relative EIVEs indices (Light, Temperature, Nitrogen) ((Dengler et al. 2023) and Disturbance Indicators (Soil Disturbance, Disturbance Severity in herb layer, Grazing Pressure)(Midolo et al. 2023)

| **SPECIES** | **FAMILY** | **STATUS** | **% TOTAL COVER** | **EIVE Light** | **EIVE Temperature** | **EIVE Nitrogen** | **Soil Disturbance** | **Disturbance Severity in herb layer** | **Grazing Pressure** |
| --- | --- | --- | --- | --- | --- | --- | --- | --- | --- |
| *Fagus sylvatica* L. | Fagaceae | Native | 9.94 | 3.004 | 4.107 | 4.861 | 0.109 | 0.122 | 0.195 |
| *Clematis vitalba* L. | Ranunculaceae | Native | 5.53 | 6.321 | 4.791 | 6.432 | 0.116 | 0.199 | 0.202 |
| *Brachypodium genuense (DC.) Roem. & Schult.* | Poaceae | Native | 4.77 | 7.079 | 4.307 | 3.617 | 0.124 | 0.276 | 0.248 |
| *Dactylis glomerata* L. | Poaceae | Native | 3.25 | 6.799 | 4.453 | 6.133 | 0.260 | 0.429 | 0.226 |
| *Quercus pubescens* Willd. | Fagaceae | Native | 2.59 | 6.513 | 5.972 | 3.868 | 0.106 | 0.144 | 0.227 |
| *Ostrya carpinifolia* Scop. | Betulaceae | Native | 2.53 | 3.776 | 6.097 | 5.263 | 0.102 | 0.119 | 0.206 |
| *Acer pseudoplatanus* L. | Sapindaceae | Native | 2.52 | 3.799 | 4.162 | 6.892 | 0.113 | 0.170 | 0.183 |
| *Ailanthus altissima* (Mill.) Swingle | Simaroubaceae | Alien | 2.33 | 7.089 | 6.073 | 7.275 | 0.280 | 0.387 | 0.194 |
| *Juglans regia* L. | Juglandaceae | Native | 2.21 | 5.968 | 5.712 | 6.663 | 0.128 | 0.197 | 0.194 |
| *Pinus nigra* J.F.Arnold | Pinaceae | Native | 2.03 | 6.970 | 5.270 | 2.229 | 0.168 | 0.166 | 0.212 |
| *Abies alba* Mill. | Pinaceae | Native | 2.01 | 3.041 | 3.682 | 4.897 | 0.107 | 0.118 | 0.195 |
| *Fraxinus ornus* L. | Oleaceae | Native | 1.99 | 5.408 | 5.904 | 3.377 | 0.101 | 0.123 | 0.216 |
| *Aesculus hippocastanum* L. | Sapindaceae | Alien | 1.69 | 4.535 | 5.229 | 6.331 | 0.130 | 0.213 | 0.186 |
| *Sanguisorba minor* Scop. | Rosaceae | Native | 1.59 | 2.954 | 4.121 | 5.918 | 0.189 | 0.452 | 0.283 |

**Table S5** The table shows the plant species representing about the 45% of the total coverage of the inland plots. The species are ordered in relation to their percentage (%) of total cover in the inland plots, their families, their status (native/alien) (Galasso et al. 2018; Bartolucci et al. 2021, 2022) and the relative EIVEs indices (Light, Temperature, Nitrogen) ((Dengler et al. 2023) and Disturbance Indicators (Soil Disturbance, Disturbance Severity in herb layer, Grazing Pressure)(Midolo et al. 2023)

| **SPECIES** | **FAMILY** | **STATUS** | **% TOTAL COVER** | **EIVE Light** | **EIVE Temperature** | **EIVE Nitrogen** | **Soil Disturbance** | **Disturbance Severity in herb layer** | **Grazing Pressure** |
| --- | --- | --- | --- | --- | --- | --- | --- | --- | --- |
| *Fraxinus ornus* L. | Oleaceae | Native | 8.92 | 5.408 | 5.904 | 3.377 | 0.101 | 0.123 | 0.216 |
| *Brachypodium genuense* (DC.) Roem. & Schult. | Poaceae | Native | 7.08 | 7.079 | 4.307 | 3.617 | 0.124 | 0.276 | 0.248 |
| *Fagus sylvatica* L. | Fagaceae | Native | 6.98 | 3.004 | 4.107 | 4.861 | 0.109 | 0.122 | 0.195 |
| *Pteridium aquilinum* (L.) Kuhn | Dennstaedtiaceae | Native | 5.32 | 5.970 | 4.215 | 3.170 | 0.129 | 0.169 | 0.210 |
| *Pinus nigra* J.F.Arnold | Pinaceae | Native | 4.95 | 6.970 | 5.270 | 2.229 | 0.168 | 0.166 | 0.212 |
| *Quercus pubescens* Willd*.* | Fagaceae | Native | 4.79 | 6.513 | 5.972 | 3.868 | 0.106 | 0.144 | 0.227 |
| *Acer opalus subsp. obtusatum* (Waldst. & Kit. ex Willd.) Gams | Sapindaceae | Native | 4.72 | 5.373 | 6.167 | 5.729 | 0.100 | 0.117 | 0.197 |
| *Quercus cerris* L. | Fagaceae | Native | 3.30 | 6.126 | 5.667 | 4.382 | 0.102 | 0.113 | 0.203 |
| *Ostrya carpinifolia* Scop. | Betulaceae | Native | 2.21 | 3.776 | 6.097 | 5.263 | 0.102 | 0.119 | 0.206 |
| *Lathyrus vernus* (L.) Bernh. | Fabaceae | Native | 1.86 | 3.468 | 4.185 | 4.714 | 0.103 | 0.120 | 0.192 |
| *Viola reichenbachiana* Jord. ex Boreau | Violaceae | Native | 1.80 | 3.429 | 4.220 | 5.550 | 0.105 | 0.134 | 0.195 |
| *Galium mollugo* L. | Rubiaceae | Native | 1.79 | 6.630 | 4.252 | 5.370 | 0.245 | 0.417 | 0.186 |
| *Rubus caesius* L. | Rosaceae | Native | 1.77 | 7.871 | 5.689 | 5.183 | 0.167 | 0.345 | 0.196 |
| *Brachypodium sylvaticum* (Huds.) P.Beauv. | Poaceae | Native | 1.74 | 4.006 | 4.384 | 5.713 | 0.109 | 0.188 | 0.199 |
| *Hedera helix* L. | Araliaceae | Native | 1.61 | 3.888 | 4.663 | 5.092 | 0.105 | 0.148 | 0.201 |
| *Cornus sanguinea* L. | Cornaceae | Native | 1.57 | 6.318 | 4.261 | 5.155 | 0.103 | 0.186 | 0.203 |


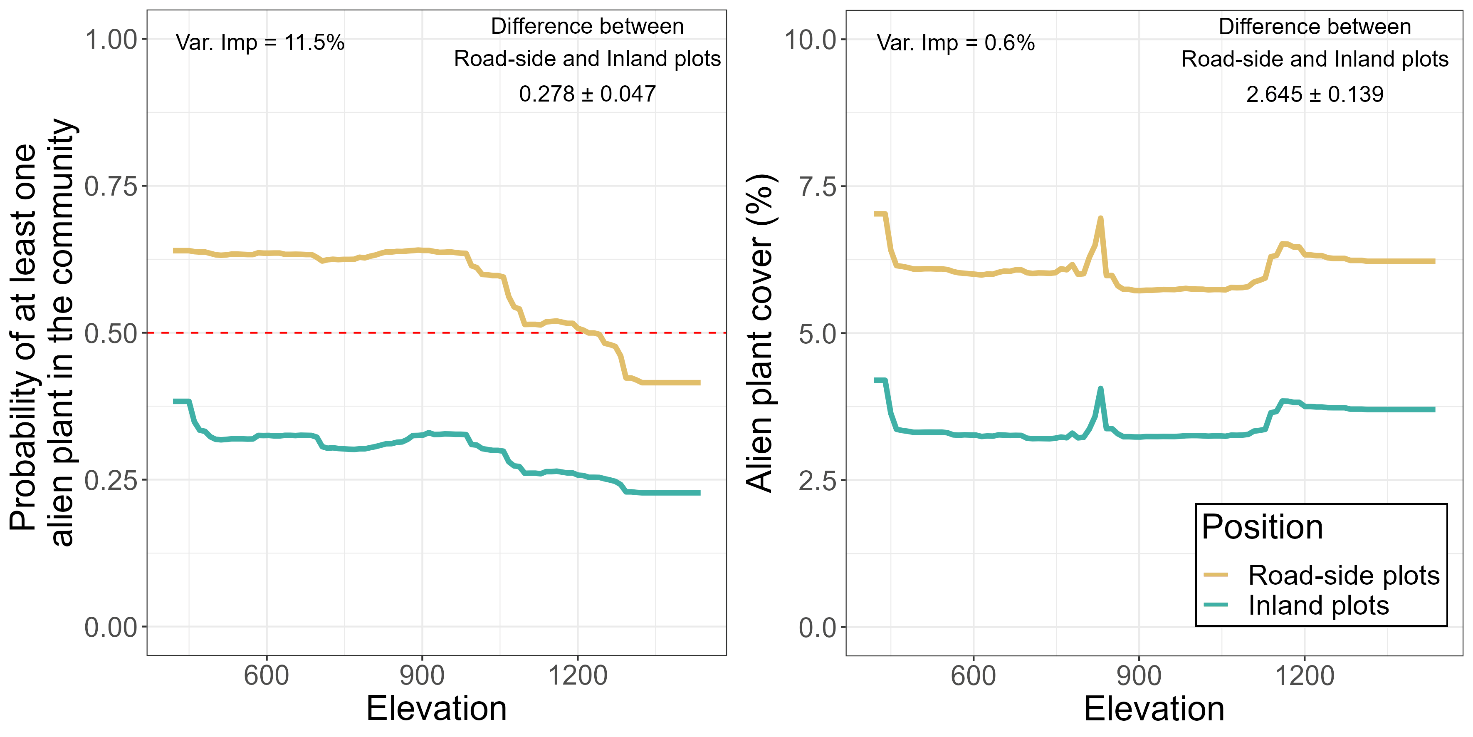


***Fig. S2*** *Partial Dependence Plots (PDP) for Elevation for the RF (Random Forest) model in the Central Apennine resident communities. For RF Classification (left hand side), the probability of at least one AP existence in the road-side or inland plots relating Elevation is shown by lines of different colours. The upper limit of the 50% probability of at least one AP presence is shown by the red dotted line. Similarly, for RF Regression, the same is shown for AP cover. The mean ± standard deviation values between the inland and road-side plots, as well as variable importance shown as Mean Decrease in Accuracy (MDA, RF classification) and the increase in mean squared error (MSE, RF regression), are displayed in both figures*


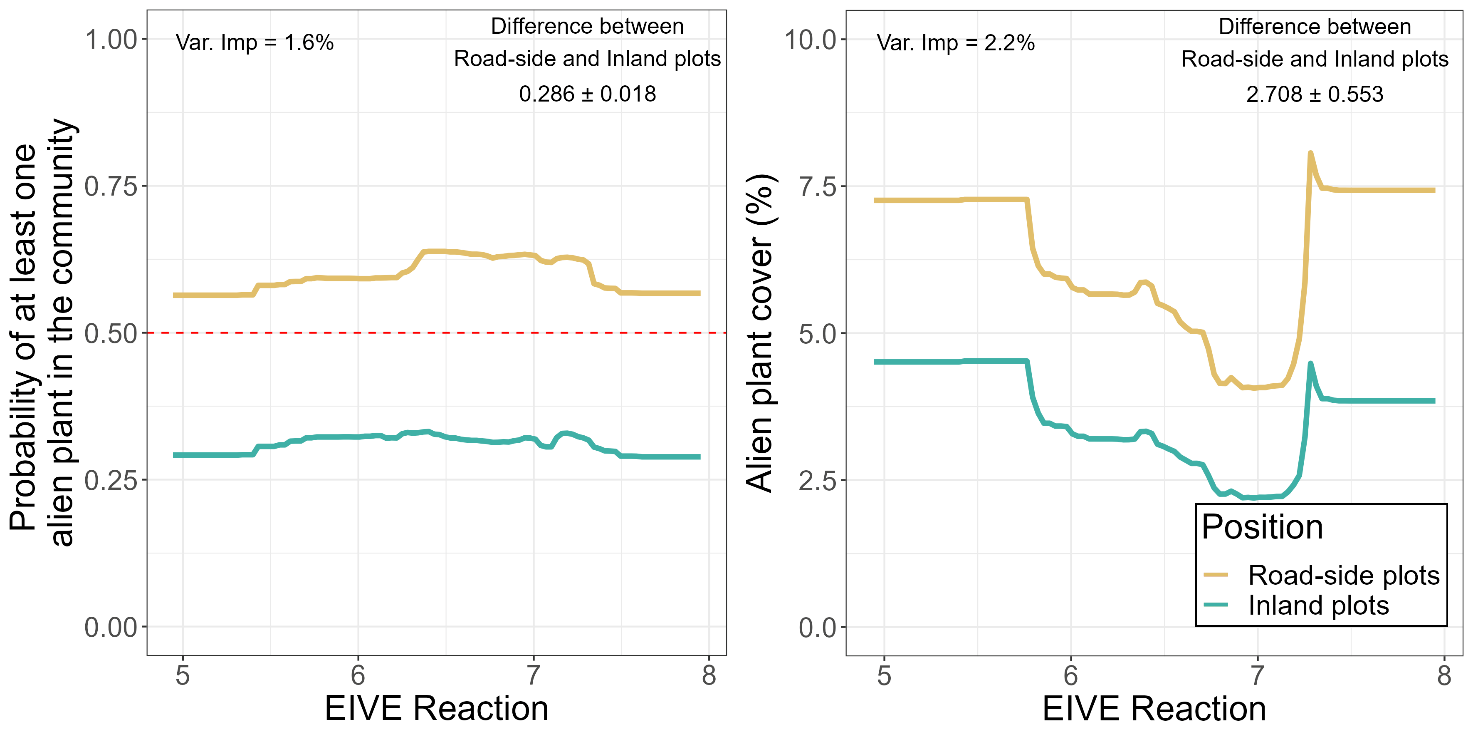


***Fig. S3*** *Partial Dependence Plots (PDP) for EIVE R for the RF (Random Forest) model in the Central Apennine resident communities. For RF Classification (left hand side), the probability of at least one AP existence in the road-side or inland plots relating EIVE R is shown by lines of different colours. The upper limit of the 50% probability of at least one AP presence is shown by the red dotted line. Similarly, for RF Regression, the same is shown for AP cover. The mean ± standard deviation values between the inland and road-side plots, as well as variable importance shown as Mean Decrease in Accuracy (MDA, RF classification) and the increase in mean squared error (MSE, RF regression), are displayed in both figures*

**REFERENCES:**

Bartolucci F, Galasso G, Peruzzi L, Conti F (2022) Report 2021 on plant biodiversity in Italy: native and alien vascular flora. Natural History Sciences. https://doi.org/10.4081/nhs.2022.623

Bartolucci F, Galasso G, Peruzzi L, Conti F (2021) Report 2020 on plant biodiversity in italy: native and alien vascular flora. Natural History Sciences 8:41–54. https://doi.org/10.4081/NHS.2021.520

Chytrý M, Tichý L, Hennekens SM, et al (2020) EUNIS Habitat Classification: Expert system, characteristic species combinations and distribution maps of European habitats. Appl Veg Sci 23:648–675. https://doi.org/10.1111/avsc.12519

Dengler J, Jansen F, Chusova O, et al (2023) Ecological Indicator Values for Europe (EIVE) 1.0. Vegetation Classification and Survey 4:7–29. https://doi.org/10.3897/VCS.98324

Galasso G, Conti F, Peruzzi L, et al (2018) An updated checklist of the vascular flora alien to Italy. Plant Biosyst 152:556–592. https://doi.org/10.1080/11263504.2018.1441197

Hammer DAT, Ryan PD, Hammer Ø, Harper DAT (2001) Past: Paleontological Statistics Software Package for Education and Data Analysis

IPBES (2023). Thematic Assessment Report on Invasive Alien Species and their Control of the Intergovernmental Science-Policy Platform on Biodiversity and Ecosystem Services. Roy, H. E., Pauchard, A., Stoett, P., and Renard Truong, T. (eds.). IPBES secretariat, Bonn, Germany. https://doi.org/10.5281/zenodo.7430682

Kassambara A, Mundt F (2020) Extract and Visualize the Results of Multivariate Data Analyses [R package factoextra version 1.0.7] https://CRAN.R-project.org/package=factoextra

Kindt R (2020) WorldFlora: An R package for exact and fuzzy matching of plant names against the World Flora Online taxonomic backbone data. Appl Plant Sci 8. https://doi.org/10.1002/aps3.11388

Midolo G, Herben T, Axmanová I, et al (2023) Disturbance indicator values for European plants. Global Ecology and Biogeography 32:24–34. https://doi.org/10.1111/geb.13603

R Core Team (2022) R: A Language and Environment for Statistical Computing. R Foundation for Statistical Computing, Vienna. https://www.R-project.org

WFO (2023) World Flora Online. Version 2023.12. Published on the Internet; http://www.worldfloraonline.org. Accessed on: 19.03.2024.  DOI 10.5281/zenodo.7460141
